# The effect of climate mitigation and adaptation policies on health and health inequalities: a systematic review

**DOI:** 10.1016/j.lanplh.2025.06.001

**Published:** 2025-07-08

**Authors:** Annika Hjelmskog, Jennifer Boyd, Amy Stevenson, Roxana Pollack, Corinna Elsenbroich, Alison Heppenstall, Jaime Toney, Jo Winterbottom, Petra Meier

**Affiliations:** a School of Health and Wellbeing, University of Glasgow, Glasgow, UK; b School of Geographical and Earth Sciences, University of Glasgow, Glasgow, UK; c Salvation Army Centre for Addiction Services and Research, Faculty of Social Sciences, University of Stirling, Stirling, UK

## Abstract

This systematic review focuses on the effects of climate adaptation and mitigation actions in high-income countries on health inequality. The paper reviews existing evidence (158 studies) on the potential of climate actions to narrow or widen health inequalities and identifies mechanisms via which these effects could occur. The extent of the evidence is highly variable and spread across multiple scientific disciplines. In some domains of climate action (such as greener transport and blue and green infrastructure) the evidence on effects on health inequality for different population groups is well developed. In other domains (such as marine conservation and biodiversity), key evidence gaps were identified. Considerable variation exists in the level of detail explaining different mechanisms. Both positive and negative effects on health inequality were found to be possible, suggesting the importance of this research area in supporting climate justice. A more coherent, interdisciplinary approach would enable robust conclusions regarding the effect of specific interventions.

## Introduction

Tackling the climate crisis is an urgent priority, with increasing concern about whether the goal of the Paris Agreement, which is to restrict the increase in global average temperature to 1·5 degrees, remains within reach.^1^ The implementation of strategies to rapidly reduce greenhouse gas emissions is currently an urgent need, particularly in high-income countries that have historically been responsible for disproportionate levels of emissions.^2^^,^^3^ In 2023, COP28 was the first Conference of the Parties to have a dedicated Health Day, thus indicating that the issue of health is rising to the fore of climate concerns, and that evidence on this topic is now an urgent need.

The climate crisis is a crucial public health issue for inequality, which has been shown to not only lead to disproportionate harms for disadvantaged groups but also to contribute to the worsening of climate breakdown itself.^4^ In addition to mitigation strategies, adaptation actions are required to respond to how the climate has already changed and will continue to change living conditions (panel 1).^5^^,^^12^ Many mitigation and adaptation actions have the potential to lead to direct population health co-benefits—for example, through improved air quality, increased active travel, and shifts to healthier, more sustainable diets.^13^^,^^14^ When such benefits are experienced among more disadvantaged populations, who stand to gain from them the most, then climate adaptation and mitigation actions could contribute to reducing health inequalities. However, such actions could also lead to adverse health outcomes. For example, increased biodiversity might introduce disease-carrying wildlife; health-promoting goods (eg, good-quality housing or food) might become less affordable, thereby exacerbating existing health inequalities; some job sectors might decline without the availability of alternative employment opportunities; and the growing financial demands of climate investment might restrict funding for other health-related public services, such as education, welfare, or health care.^15^ The literature on this topic is fragmented and often siloed, considering the effect of only one type of mitigation or adaptation action, with research spread across numerous scientific disciplines.

Panel 1 **Climate mitigation and adaptation policy actions commonly implemented in (or advocated for) high-income countries can be categorised under the following broad headings5**

**Mitigation** ^6^ ^,^ ^7^

• Reducing energy consumption (eg, implementing programmes to improve energy efficiency in homes and applying carbon pricing)
• Promoting renewable energy policies (eg, subsidies for businesses to use renewable energies and disincentives for use of industrial fossil fuel)
• Developing and preserving carbon sinks (eg, supporting woodland and peatland carbon projects)
• Promoting low-emission transport (eg, encouraging the use of low-emission vehicles and active transport methods)
• Improving waste management (eg, implementing measures to increase recycling or reuse and reduce harmful disposal)
• Promoting sustainable land use (eg, supporting forestry and reducing emissions through agriculture [such as through lower fertiliser use])

**Adaptation** 8, 9, 10, 11

• Preparing for or responding to flooding and coastal change
• Responding to higher temperatures
• Managing natural resources, including water
• Improving infrastructure resilience
• Boosting agricultural resilience and food security
• Monitoring and responding to emerging pests and disease

The potential for climate policies and actions to affect health and health inequalities has been suggested in previous academic works, reports, and strategy documents produced by and for international policy makers.^16^, ^17^, ^18^, ^19^, ^20^, ^21^, ^22^, ^23^ Although the 2023 and 2024 *Lancet* Countdown reports have found little evidence of progress towards using climate interventions to generate positive health effects,^24^^,^^25^ in some areas, the evidence base is fairly advanced. For example, in terms of mitigation policy, the improvement of home insulation can lead to better thermal comfort, improved mental health, and reduced incidence of respiratory and cardiovascular disease.^21^ In 2023, the *Lancet* Pathfinder Commission also published estimated health co-impacts for mitigation actions that lead to reduced air pollution; higher consumption of healthy, sustainable diets; and increased use of active and public transport.^26^ As for adaptation, the risks associated with failing to adapt to extreme events such as flooding (eg, by taking out flood risk insurance) or increasing incidences of heatwaves and other extreme weather events have multiple implications on health.^27^^,^^28^ However, a thorough discussion and evidence of the possible effect on health inequalities, or the specific effect on vulnerable populations, is largely unavailable.^29^^,^^30^ In addition, the prevailing emphasis of governments and scientists regarding the outcomes of sustainable development is more on economic, social, and environmental sustainability than on human health.^31^^,^^32^

Such research is important because climate change and its impacts are not felt equally, and inequalities are experienced within countries as well.^33^ The response to climate change (through both adaptation and mitigation), therefore, needs to take this inequality into account. The effects of climate change on health are experienced along existing lines of inequality,^34^ and policy responses have the potential, when consciously designed, to minimise or prevent unequal effects. Without such intentional design, however, adaptation and mitigation measures might miss valuable opportunities to reduce or reverse health inequalities, or in some cases, inadvertently widen health inequalities. Decision makers need to better understand and respond to the effects of climate action on health inequality, to adopt climate policies that have the greatest potential for offering co-benefits for health and health inequality, and to account for or mitigate possible negative unintended consequences.

Existing reviews have explored the effects of climate mitigation and adaptation measures on inequalities without specifically considering health inequalities,^35^^,^^36^ or have assessed the effects on health without considering the equitable or inequitable distribution of these effects.^20^^,^^26^^,^^36^^,^^37^ Therefore, there is a need for a comprehensive synthesis of the evidence, specifically in relation to the effects of climate mitigation and adaptation measures on both health and health inequalities. A 2023 scoping review highlighted the need for a better understanding of the implications of these policy areas on health inequality.^20^ In this Review, we seek to fill this gap in the research literature by reviewing evidence dispersed across disciplines, recognising that many diverse climate policy action areas are expected to be crucial for both health and health inequalities, but have yet to be comprehensively considered as a coherent and unified issue. We also aimed to summarise the types of climate mitigation or adaptation framed as affecting health and health inequalities; to identify the types of health and health inequality outcomes and the measures used to capture these outcomes; to explore the mechanisms between climate action and health inequalities that have either been tested or proposed; and to understand the climate mitigation or adaptation actions that are not considered in the context of health and health inequality.

This Review is the first to provide a comprehensive synthesis of what is currently known across a breadth of climate actions and health equality outcomes. Although research on this topic is relevant globally, this Review focuses on high-income countries, to pay close attention to the within-country health inequalities, and the health injustices experienced between different population groups. In high-income countries, the most disadvantaged groups are the ones that have contributed least to causing the climate crisis, but are most vulnerable to its effects; therefore, the questions of equitable adaptation and mitigation responses are of key importance in this context. The primary aim of this Review is to gain a better understanding of the relationships between these types of policy actions and health and health inequalities in high-income contexts.

## Methods

### Search strategy and selection criteria

The protocol for this Review was preregistered on PROSPERO (CRD42022361557)^38^ and the Review followed PRISMA guidelines.^39^ The search window for this study was from Jan 1, 2000, to Dec 31, 2023. The Web of Science, MEDLINE, and Scopus databases were searched to identify peer-reviewed literature that explored the effect of climate mitigation or adaptation action(s), or both, on health and health inequalities. The full search strategy is available in appendix 1 pp 1–7. All authors had access to these databases via University of Glasgow library accounts.

The PECOS criteria for inclusion are listed in panel 2. Full-length peer-reviewed papers published in English and those that explicitly explored the relationship between one or more climate mitigation or adaptation action(s), or both, one or more health outcome(s), and one or more health inequality measure(s) were included. We restricted our Review to studies set in high-income Organisation for Economic Cooperation and Development (OECD) countries as classified by the World Bank^40^ and studies carried at the global scale in which most research took place on OECD high-income countries. Existing systematic reviews and meta-analyses were included, as extracting the mechanisms between policy actions and health inequalities from these studies is possible. Books, editorials, discussion papers, letters, and non-peer-reviewed publications were excluded.

Panel 2 **PECOS criteria for study inclusion**

**Population**

Office for Economic Cooperation and Development in high-income countries only∗

**Exposures**

Any climate mitigation or adaptation actions (responses to climate change aimed at reducing emissions or adapting to the changing climate)

**Comparisons**

Comparison of socioeconomic position (any measure, including area-level deprivation and individual measures [eg, income, education, sex, ethnicity, disability, and age]) wherever possible, to understand any existing health inequalities

**Outcomes**

Any physical health outcome (eg, mortality, morbidity, or healthy life expectancy), wellbeing or mental health outcome (eg, quality of life or depression), and health behaviour (eg, physical activity or diet)

**Study design**

Inclusion of empirical studies: cross-sectional or longitudinal, reports produced by agencies and organisations (when peer reviewed), or both

Exclusion of books, editorials, discussions, letters, commentaries, and non-peer-reviewed publications

∗Australia, Austria, Belgium, Canada, Chile, Czech Republic, Denmark, Estonia, Finland, France, Germany, Greece, Hungary, Iceland, Ireland, Israel, Italy, Japan, South Korea, Latvia, Lithuania, Luxembourg, the Netherlands, New Zealand, Norway, Poland, Portugal, Slovakia, Slovenia, Spain, Sweden, Switzerland, the UK, and the USA

An initial list of climate mitigation and adaptation actions was developed from an analysis of relevant policy documents from the UK and its devolved nations, as an example of policy spaces applicable to high-income countries,^5^ and cross-checked against several international examples from high-income countries.^7^^,^^10^^,^^11^^,^^41^ An extensive list of search terms was used to capture themes of climate change (appendix 1 pp 2–4, 6). Terms were slightly adapted depending on database requirements. Additional papers to be included were identified from the reference lists of papers that were returned after database searching.

All records were imported to Rayyan and duplicates were removed. Titles and abstracts were screened to identify records that matched the inclusion criteria. Full-text screening was then carried out to decide inclusion. Screening was carried out by four reviewers (JB, AS, AHj, and JW). Over one-fifths of the screened papers (n=108 [20%] of 524) were double screened to validate that the papers were included correctly. No disagreement happened between the reviewers regarding inclusion.

### Data analysis

Data from the papers were extracted by five reviewers (JB, AS, AHj, RP, and JW). The accuracy of data extraction was independently assessed by a second reviewer (JB, AS, AHj, or RP) for 32 (20%) of the 158 papers. In the case of disagreement, a minimum of three reviewers (JB, AS, and AHj) referred to the paper in question and a consensus was reached.

A data-extraction matrix was developed, which included study characteristics (publication year, location, design, and aim); information about the mitigation or adaptation action, or both (description and category); measures (health outcome and inequality measures); and effects (direction of effects of the action on health and health inequality, in addition to the effects of tested or hypothesised mechanisms). Mechanisms were any described processes through which the effects of mitigation or adaptation actions on health and health inequalities could be identified from the empirical results or from the text as proposed by authors. The text as proposed by authors was extracted to reflect the authors’ reasoning and use of language.

Descriptive summary statistics were used to describe search results and study characteristics. To understand the mechanisms underlying the effect of these actions on health inequalities, actions were grouped into 16 broad domains, aligning with the preliminary typology:^5^ biodiversity; business and industry; marine conservation; waste; river and coastal floods and erosion; natural carbon sinks and land conservation; extreme weather events; water availability and quality; forestry; biosecurity and communicable disease; agriculture, food systems, and land use; buildings and infrastructure; temperature changes; greener transport; blue and green infrastructure; and cleaner energy and reducing emissions. Actions within domains were also grouped into intervention types and the connections between the action and health inequality, including the direction of the effect, was recorded.

## Results

**Figure 1:**
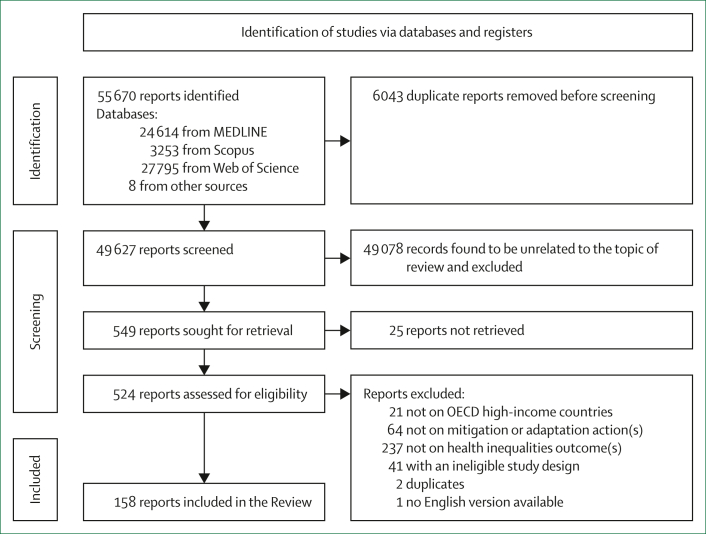
PRISMA flow diagram OECD=Organisation for Economic Cooperation and Development.

Searching of electronic databases returned 55 670 records. An additional eight records were identified as clearly relevant papers upon going through the reference lists of papers that were selected for inclusion after database searching. The total number of records reduced to 49 627 following the removal of duplicates. Title and abstract screening led to the selection of 524 records for full-text screening, and 158 of these met the inclusion criteria for data extraction (figure 1). Papers that did not meet the criteria outlined in panel 2 were excluded. Attempts to retrieve inaccessible records were made through the search databases, University of Glasgow Library services and Google Scholar.

The descriptive characteristics for the included studies are given in appendix 2 (pp 1–68). Many papers (n=33) described studies with a global scale or studies based across multiple countries, rather than focusing on one particular country. Some papers (n=8) also focused on Europe as a continent. Of the country-specific papers, those from the USA accounted for the highest proportion (n=44), with fewer papers from the UK (n=14), Australia (n=12), New Zealand (n=7), and Germany (n=6). Five or fewer papers originated from each of the following countries: Canada (n=5), Denmark (n=1), Italy (n=5), Poland (n=1), Finland (n=1), Spain (n=5), Sweden (n=4), Switzerland (n=1), the Netherlands (n=2), Greece (n=1), and Norway (n=1). Notably, some papers originated from OECD high-income countries in Asia; these included papers from only Japan (n=6) and South Korea (n=1).

The selected papers also included many reviews (n=62). However, of the reviews, only a small proportion implemented rigorous review techniques: systematic reviews (n=8), scoping reviews (n=4), and meta-analyses (n=1). Existing systematic reviews focused solely on one type of climate mitigation or adaptation action per review (eg, air pollution control strategies,^42^ urban agriculture,^43^ and mitigation policies on the transport sector).^44^ 40 papers were original research studies, with study designs that included cohort, cross-sectional quantitative, case-study, natural experiment, policy analysis, programme evaluation, qualitative, and mixed-method designs. Of the remaining 56 papers, 36 reported modelling studies, which included epidemiological modelling typically estimating health harms using risk functions (eg, for level of air pollution), with a few (n=3) implementing simulation modelling techniques. Health impact assessments (n=6), reports (n=6), theoretical work (n=4), and papers describing conceptual frameworks (n=4) were also included.

**Figure 2:**
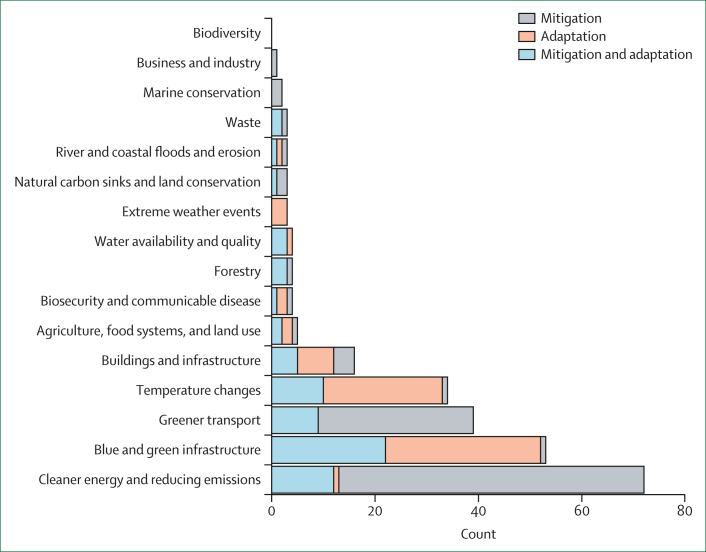
Distribution of papers across climate action categories that focused on mitigation, adaptation, or both

The included papers covered a wide range of climate mitigation and adaptation actions, with the most commonly considered actions being cleaner energy and reducing emissions; blue and green infrastructure; and greener transport (figure 2). Only 15 papers considered the effect of action on health inequalities in the domains of business and industry (n=1); marine conservation (n=2); waste (n=3); river and coastal floods and erosion (n=3); natural carbon sinks (n=3); and extreme weather events (n=3). Most papers focused only on the effect of climate mitigation action (n=71), whereas others looked at both mitigation and adaptation action in combination (n=51), and some focused only on adaptation action (n=36). The thematic distribution of these papers is given in figure 2.

Studies looked at various health outcomes, including physical health (eg, all-cause mortality, respiratory effects, cancer, cardiovascular disease, stroke, disability-adjusted life-years); mental health and wellbeing; and health behaviours (eg, physical activity [such as walking and cycling] and dietary behaviour [such as eating foods with lower greenhouse gas-emission effects]; detailed in appendix 2 pp 1–68). Most studies (n=104) found positive co-benefits of climate mitigation and adaptation action on health, 11 papers found negative effects, 25 papers found mixed (or both positive and negative) effects, and the remaining 18 found no or negligible effects.

The table summarises the included climate mitigation or adaptation actions and the evidence relating to their effects on health inequality, grouped by domain (as per the initial typology).^5^ Some interventions span more than one domain; for example, decarbonisation initiatives that include electrification of public transport will be classified as both greener transport and cleaner energy and reducing emissions. Additionally, measures for reducing air pollution have crucial implications for health, but are a feature (or outcome) of several existing domains, rather than being classified separately.

**Table:** Summary of evidence

	Summary		
**Biodiversity** (n=0 studies)	No studies examining the effects of climate adaptation or mitigation measures on health inequality were identified in this domain.		
**Biosecurity and communicable disease** (n=2 studies: A=1 and M and A=1)	Positive (n=1) Negative health effects of threats such as foodborne pathogens (increasing due to climate change) for the groups with the highest risk can be minimised through targeted surveillance and protection risk.	··	Neutral (n=1) Behavioural and technical responses to health threats are potentially inadequate in addressing the long-term structural causes of increased vulnerability of some disadvantaged groups (eg, socioeconomic deprivation or existing clinical vulnerability) to climate change-induced health threats.
**Buildings and infrastructure** (n=16 studies: A=4, M=7, and M and A=5)	Positive (n=14) Energy efficiency measures in homes offer the largest health benefits (via temperature and air quality control) to low-income, indigenous, older (≥65 years), or frail populations, in addition to reducing negative health consequences for those in fuel poverty. Health inequality improvements are the most effective when the energy efficiency interventions are accompanied by sufficient ventilation measures, allowing a greater number of poor or vulnerable households to meet their basic needs and support thermal comfort. Minor positive effects on childhood asthma are identified from retrofit actions. Benefits are identified from targeted urban design that delivers health protection infrastructure for vulnerable groups (eg, spaces for social connection, cooling, and physical recreation). Targeting the location of this infrastructure has key health effects for low-income households.	Negative (n=2) Some individual home adaptation measures are expensive, and therefore, the health benefits are more accessible to affluent groups (eg, biophilic design). Vulnerable groups have increased restrictions to their adaptive capacity. Home ventilation measures can increase the exposure to pollutants for some low-income populations living near sources of outdoor air pollution. Some regulatory improvements to infrastructure (eg, in workplaces and office buildings) can widen health inequalities between those who work in formal versus informal (exposed) settings.	Neutral (n=1) One study in this domain noted that the observed effect on health inequalities was small. A general paucity of research on the effects of other large infrastructure projects (eg, railways, airports, roads, and bridges) on health inequality was identified.
**River and coastal floods and erosion** (n=3 studies; A=1, M=1, and M and A=1)	Positive (n=2) The value of nature-based flood solutions (reduced mental stress, increased use of green urban infrastructure for physical activity and recreation) is majorly experienced in deprived neighbourhoods. However, some caveats remain. For instance, policies to address health inequality will be more effective when aimed at protecting against consequences such as gentrification (eg, by providing affordable housing).	Negative (n=1) Managed retreat affects indigenous and low-income populations disproportionately.	··
**Blue and green infrastructure** (n=35 studies; A=4, M=1, and M and A=30)	Positive (n=22) Green infrastructure that is strategically targeted towards disadvantaged neighbourhoods or specific population groups can lead to the biggest health gains for vulnerable or disadvantaged populations, through improved mental health, reduced stress, increased physical activity, and the cooling effects of greenery. Measures such as urban parks and forests, or green roofs on buildings with older residents can reduce heat-related mortality for vulnerable groups and the urban heat island effect. Targeting areas of high urban density, low-income neighbourhoods, or schools, can have a positive effect on health and mitigate long-term health inequality, such as redlining policies in the USA. School-based green spaces offer the potential to improve health for children from low-income families who are increasingly growing up in urban areas with restricted access to nature. Interventions are more effective at reducing health inequalities when implemented with proper foresight, regulation, and community buy-in, in addition to protecting against green gentrification effects (such as providing affordable housing).	Negative (n=6) Several studies (n=5) noted the strong correlation of socioeconomic status with the restriction or prevention of reductions in health inequalities. Benefits of increased green spaces are often experienced by affluent or privileged groups. Neighbourhoods become more desirable as a result of green and blue infrastructure, leading to gentrification, and health benefits are experienced by those who can afford to move into such areas. Actions that benefit one target group (such as children’s health benefits from green schoolyards) could exclude others, as green infrastructure is noted to be more common in privileged districts. Some cases of increased allergens are worse in children and young individuals (13–18 years).	Neutral (n=3) Studies that identified no effect on health inequalities found that the greening interventions do not disrupt existing lines of inequality. Socioeconomic status attenuates the effects of green spaces on health. Additionally, studies noted insufficient focus on vulnerable groups with the most to gain (and their needs). Other interpretations of neutral effects consider community-level greening interventions to be inadequate to tackle the structural root causes of systemic health inequalities.
**Clean energy and reduced emissions** (n=72 studies; A=1, M=59, and M and A=12)	Positive (n=43) Most studies (n=25) on decarbonisation actions (reducing the use of fossil fuel energy, replacing fossil fuel energy with renewable sources, or both) found positive effects on health inequality, noting that reduced combustion of fossil fuels, leading to lower levels of toxicity and climate-altering pollutants in the air, could have larger health effects on vulnerable populations who are typically disproportionately exposed or affected (eg, children and infants, indigenous people). Decommissioning fossil fuel power plants benefits the health of the low-income, rural, and marginalised populations. Some papers (n=2did note variability in the effects of different decarbonisation scenarios on health inequality; a study on decarbonising light duty vehicles found better health effects for Hispanic groups and people of colour than an alternative scenario decarbonising industry. Actions that improve air quality by reducing wood-burning activities improve the health outcomes for those with a low socioeconomic status. Building-level or small-scale installation of renewable energy sources (eg, solar photovoltaic) benefits vulnerable groups (eg, those with asthma, older individuals, and people with low income). Few studies (n=2) on financial carbon policies (eg, carbon taxes) report positive health effects for minority groups, either through improved air quality or incentives to increase levels of physical activity (eg, for children). Studies considering reducing emissions within the health sector found benefits from an e-health (telemedicine) intervention that brought medical care to remote and underserved communities and predicted benefits for children’s health from fossil fuel divestment. A reduction in health inequalities for some groups is possible via mitigation measures such as not using air conditioning, as air conditioning is mostly used by affluent households; however, this measure might lead to negative health outcomes overall.	Negative (n=27) Some studies (n=3) on decarbonisation actions found negative effects on health inequality; however, the scenarios considered were not adequate (neither far reaching nor targeted enough) to reduce negative health effects for the populations in need (ie, leading to continued or exacerbated spatial or racial health injustice). Some negative effects on health inequality were observed for large-scale renewable infrastructure projects, including wind turbines and hydropower dams, with possible displacement of some marginalised and rural communities, in addition to increasing noise pollution (although, of note, marginalised and rural communities are also more likely to live near fossil fuel power plants and experience the negative health effects of these plants). Most studies (n=6) on financial carbon policies (eg, carbon pricing, carbon trading or auctions) found them to be regressive and have negative health effects on poorer groups, by raising the prices of products and services inequitably, or by allowing carbon offsets that either create a burden for vulnerable people or reflect missed opportunities to improve their health outcomes (eg, continued emissions from power plants that might otherwise be commissioned). Not using air conditioning has negative consequences for some groups, from heat-related illnesses (eg, older individuals who are at a greater risk of stroke-related mortality). Some barriers to accessibility (eg, financial) are identified for household-level renewable energy projects (eg, solar photovoltaic). With a couple of exceptions, most studies (n=8) on dietary change (switching to diets with reduced effects on greenhouse gases) found negative effects on health inequality, noting disadvantages due to sex (primarily a greater health benefit identified for men), age, and socioeconomic status. The higher cost of healthy diets with low planetary effects is considered a barrier for low-income groups to adapt to dietary change, and this inequality could increase as some foods become less available or affordable.	Neutral (n=13) Some papers (n=9) noted no effect on health inequalities (and therefore, some missed opportunities) from carbon emission reductions and carbon offsets or cap-and-trade programmes for the older or low socioeconomic status groups. Several studies in this domain (n=4) did not analyse the effects of clean energy and reduced emissions on health inequality in detail but rather noted that socioeconomic status should be taken into consideration. Other studies showed that effects on health inequalities are context-dependent, and that generalisable patterns are not necessarily clear. These studies also noted the potential for positive (or negative) effects on health inequality, but these effects will not necessarily be realised (context, manner of implementation, and effective targeting will influence these outcomes).
**Green transportation** (n=39 studies; M=30 and M and A=9)	Positive (n=27) Green transportation (low-emission or zero-emission vehicles, active travel, and public transport) can improve air quality and street safety, reduce noise pollution, and enhance the quality of life. These effects are experienced spatially, and in some cases, can provide the largest health benefits to those who have the most to gain, suggesting a narrowing of health inequalities. For instance, marginalised communities with higher rates of cardiovascular disease, diabetes, and stroke will benefit more from adopting active travel measures, and reducing motorised transport can reduce negative air quality exposure in higher-density, urban communities. Children can benefit from green transportation through reduced air quality-related visits to emergency departments and increased outdoor play on quieter, safer streets. Walking and cycling are potentially affordable to those with low incomes; hence, interventions aimed at improving the quality and accessibility of active travel infrastructure could reduce health inequality. Optimising public transport routes and frequencies to meet the needs of vulnerable individuals can help to avoid the possible negative effects of increased public transport use on health inequality.	Negative (n=12) One study reported an increase in exposure to health harms (eg, pollution, extreme heat) when waiting at or travelling to public transit stops, with larger effects on vulnerable populations. Active travel is found to have several accessibility barriers (social, financial, and cultural), which can exclude disabled people, ethnic minorities, and those with low incomes. Some segregated or isolated active travel routes are also less likely to be used by women. Electric vehicles can exacerbate health inequalities along socioeconomic lines, being inaccessible to low-income groups. Although some air quality benefits from electric vehicle use might be felt by all—eg, in busy urban areas, there is a risk that the power needed for electric vehicles (if not clean, renewable energy) displaces the pollution and health harms to those living near power-generating facilities, which are often rural, disadvantaged communities. The use of electric vehicles might also perpetuate spatial injustices caused by road traffic—such as segregation—that negatively affect the wellbeing of young, old, and disabled people.	Neutral (n=9) Studies that found no effect on health inequalities (eg, through the effect of improved air quality) suggested that the existing levels of inequality could persist due to an insufficiency of effective targeting, possibly through a failure to ensure that low-emission policies are indeed affecting the neighbourhoods that need them, or a scarcity of measures to remove the cost barriers to lower-emitting modes of transport.
**Temperature changes** (n=34 studies; A=23, M=1, and M and A=10)	Positive (n=21) Vulnerable groups (ie, older individuals and children) gain more from adaptation measures and programmes (eg, heat action plans) that are specifically designed to target or monitor these populations. Air conditioning reduces heat-related illness and mortality for vulnerable groups, in addition to social interventions or community outreach that offer support for marginalised or isolated groups. When sufficiently inclusive, community resources such as cooling centres in public spaces can also reduce heat harms for vulnerable populations, such as those who are homeless.	Negative (n=13) Some studies (n=6) found that the current distribution of cooling measures highlights the health inequalities that they perpetuate (eg, use of air conditioning, which correlates with deprivation and represents strong levels of social disparity). The use of air conditioning is more common in affluent households, giving this group higher levels of protection against heat. Air conditioning also creates additional anthropogenic heat and contributes to pollution via its power sources (unless the source of power is completely clean electricity). This pollution (eg, from power plants) is more likely to cause negative health effects for low-income rural residents who live in the vicinity. Negative health effects of air conditioners (eg, circulation of pathogens and allergens) has a potentially disproportionate effect on vulnerable individuals with respiratory problems.	Neutral (n=5) Some interventions did not result in the expected health inequalities; studies in indigenous populations showed that physiological and sociocultural adaptations could offer similar levels of heat protection as those from the technological or infrastructural adaptations favoured by non-indigenous groups.
**Forestry** (n=4 studies; M=1 and M and A=3)	Positive (n=3) Targeted afforestation in low-income or periurban areas, where residents are more likely to be exposed to the surface urban heat island effect, can provide proportionally large health benefits to disadvantaged groups (eg, low-income, older populations).	Negative (n=2) A study that analysed existing sociospatial distributions of urban forests found that neighbourhoods with a higher number of older residents and low land prices have access to the smallest amount of cooling resources. Of note, the design or implementation of such interventions is crucial; failing on these accounts can result in a risk of replicating or exacerbating the existing health inequality.	··
**Agriculture, food systems, and land use** (n=5 studies; A=2, M=1, and M and A=2)	Positive (n=5) Low-income populations tend to have reduced access to urban agriculture but could benefit the most from the same, especially from community gardens to reduced food scarcity. Urban agriculture can revitalise low-income communities. Reducing greenhouse gas emissions from the agriculture sector can have widespread population health benefits (eg, through improved air quality) that could also reduce health inequalities when the interventions are targeted to benefit areas of high deprivation.	Negative (n=1) One study noted that well-placed, socially advantaged people benefit the most from urban food policies to support urban farming.	··
**Water availability and quality** (n=4 studies; A=1 and M and A=3)	Positive (n=1) Better management of drinking water supplies as a response to extreme heat events can protect vulnerable groups (eg, older individuals) from dehydration.	Negative (n=3) Using untreated water (eg, for cleaning or food growing) can be even more challenging to manage in remote communities, where safe water supplies are already scarce. A study on water storage adaptation for horticulture found increased negative health effects for exposed indigenous women workers.	··
**Natural carbon sinks and land conservation** (n=3 studies; M=2 and M and A=1)	Positive (n=3) Effects on health inequality could be positive, depending on the method of implementation.	Negative (n=2) Effects on health inequality could be negative, depending on the method of implementation.	··
**Extreme weather events** (n=3 studies; A=3)	Positive (n=2) Tailored advice and support (such as written or printed communications, stockpiling of medicines, priority access to electrical backups, community emergency shelters) for populations at risk during extreme weather events can mitigate the risk of exacerbated health adversities within vulnerable groups.	Negative (n=1) Forecasts and disaster alerts that overestimate the severity of weather events such as hurricanes can lead to larger negative consequences for vulnerable populations than the disaster itself (eg, through cancellation of health-care appointments for pregnant women and infants).	··
**Waste** (n=3 studies; M=1 and M and A=2)	Positive (n=2) Community-level mitigation actions (eg, swap shops and food waste reduction) have positive effects on the local population (with restrictions).	Negative (n=1) Interventions such as reducing food waste can be individualistic. Vulnerability reduces adaptive capacity; therefore, interventions that do not take this vulnerability into account increase the risk of exacerbating health inequalities.	Neutral (n=2) Actions at the community level are potentially inadequate for tackling the root causes of social inequalities. Another study that listed waste in its interventions noted the importance of targeting low-income households to attain greater health effects.
**Marine conservation** (n=2 studies; M=2)	Effects on health inequality are not described in detail for this domain.		
**Business and industry** (n=1 study; M=1)	Effects on health inequality are not described in detail for this domain		

A=only adaptation strategies. M=only mitigation strategies. M and A=mitigation and adaptation strategies.

Mechanisms related to the effect of interventions on health inequalities were not considered for all domains (a notable evidence gap exists for biodiversity), and within individual papers, mechanisms were sometimes only specified for a few of the full range of domains a study covered. Overall, wherever effects on health inequalities were observed, the evidence was mixed regarding whether a lessening or widening of health inequalities is expected. Some adaptation or mitigation measures are argued to reduce health inequalities when they target issues that particular groups are more exposed to (eg, energy efficiency measures in homes) and those addressing the housing conditions that commonly affect low-income groups, children, or older adults.^45^^,^^46^ However, interventions that are deemed to be more expensive to use or install—including home improvements such as the use of air conditioning^47^ or switching to electric vehicles^48^—are most likely to be inaccessible to disadvantaged groups (unless subsidised accordingly), risking a widening of health inequalities. The mixed evidence is primarily associated with the targeting and implementation of any given intervention, which decides who is most likely to be exposed to potential risks and who is most likely to benefit.

When considering health inequalities in combination with general health effects (for the general population), we found that a substantial number of studies (n=56) observed positive effects for both. These win-win scenarios suggest that in many cases, both improving the aggregate health outcomes and reducing the health inequalities is possible; for example, decarbonising the energy sector was shown not only to avoid deaths at the population level but also benefit those in poverty the most, given that they are exposed to pollution most often.^49^ Several studies (n=26) found positive effects on general health but a negative effect on inequality. These win-lose scenarios are of particular concern as they suggest a continuation or exacerbation of climate injustice within these high-income study contexts. For example, one study found that cooling resources such as forests and cooling centres are unequally distributed; therefore, while the resources could improve health at the population level, they exacerbate health inequalities, as the older populations (>65 years) and low-income areas have less access to these resources.^50^ A smaller number of studies (n=10) recorded lose-lose effects, wherein the overall health outcomes were negatively affected and health inequalities increased as well. For example, a study that looked at the effect of a water storage project for horticulture found that the project in fact led to greater exposure to intensifying hot, dry, and wet conditions and dust, worsening health outcomes, particularly for Māori women.^51^ The remaining studies (n=66) found more mixed results, often reporting contextual factors as caveats or confounding factors, or as is particularly the case for review articles, reaching multiple conclusions for the various interventions considered.

The overall treatment of health inequalities within this literature can be viewed as either active or passive. The extent to which health inequalities were either actively or passively featured within the studies varied substantially, with some papers treating effects on health inequality as the main focus,^52^ and others only mentioning health inequalities as a footnote, or as a secondary or even tertiary concern.^19^^,^^53^ Although many studies considered the evaluation of health inequalities as crucial components of their study design, several made more casual observations of the relationship between health outcomes and different population vulnerabilities, or simply stated that social status or inequalities should be taken into account (despite not doing so themselves in these particular studies).^54^^,^^55^

The climate mitigation and adaptation actions themselves could also have either an active or a passive effect on health inequalities. An active intervention could be one that specifically targets population groups that have more to gain than the general population (such as heat response plans that are specifically designed for older people).^56^ Passive interventions, in contrast, included more general mitigations or adaptations, such as sector-wide decarbonisation or transport strategies.^57^^,^^58^ The interpretation of interventions within the same domain can also vary (eg, urban greening), and some studies predict or observe a narrowing of health inequalities due to the larger gains available for disadvantaged groups,^59^ whereas others conclude that health inequalities either continue or get wider along existing axes of disadvantage.^60^

The ways that studies report on the mechanisms relating to their health inequality conclusions vary, with a large range observed in the level of detail considered. An example of a well evidenced but context-dependent health inequality mechanism is the use of air conditioning:^47^^,^^61^^,^^62^

An increase in extreme temperatures leads to an increase in the use of air conditioning by those with access, which in turn reduces the discomfort from or exposure to high temperatures. The reduced exposure to high temperatures reduces heat-related illnesses, including heatstroke mortality, leading to fewer heat-related deaths or illnesses among groups at risk of heat-related health effects (eg, older people or children), which in turn reduces the health inequalities by age. However, increased use of air conditioning causes waste heat and possible greenhouse gas emissions at the power source, which leads to increased anthropogenic heat and the urban heat island effect. This effect will increase future summer maximum temperatures, which increase the exposure of some groups (eg, low-income groups) to extreme heat. Such exposure will cause more heat-related deaths each year, particularly for those who cannot afford air conditioning, thus ultimately increasing the health inequalities by socioeconomic status.

Conversely, defined mechanistic detail regarding the effect of interventions within the natural carbon sinks and land conservation domain on health inequality remains insufficient. The three studies that did include carbon sinks within their climate actions were all review papers that considered a broad range of emission-reducing interventions. Two of the three papers noted that effects on health inequality are dependent on the manner and context of implementation: how interventions are carried out and for whom.^13^^,^^36^ The literature reviewed in this study, however, does not detail the health inequality mechanisms for natural carbon sinks and land conservation, highlighting an evidence gap.

In studies that did not fully investigate the mechanisms leading to health inequalities, the patterns reported could possibly reflect a reverse causation, wherein population groups with the means and opportunity to do so are choosing to live in places with higher levels of infrastructure that both support good health and offer protection against the harms of climate change. One such example is the relationship between natural capital and affluent neighbourhoods.^63^

**Figure 3:**
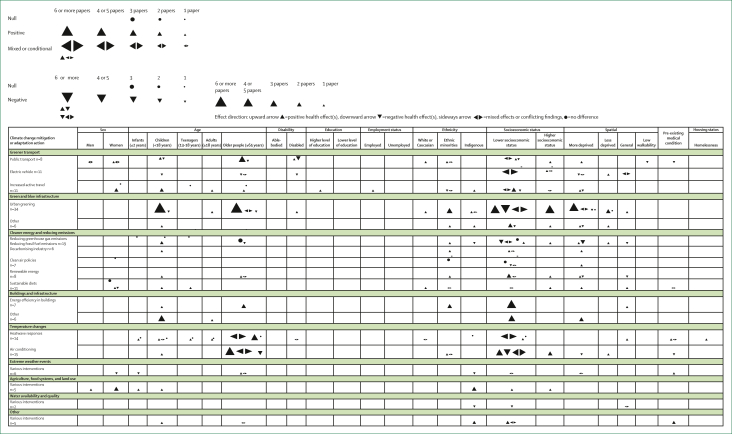
Effect direction plot of the effect of climate mitigation or adaptation actions on different health inequality characteristics

When exploring health inequalities, papers included in this Review tended to focus on socioeconomic, age, and spatial differences, with fewer papers focusing on inequalities in health related to ethnicity, employment, education, sex, and disability (figure 3). However, many of these studies did not report comparisons of effects on different groups. Instead, several investigated the effects of action on particular disadvantaged populations only (eg, children, adults older than 65 years) or merely explained why targeting particular groups with action is important to ensure that health inequalities are not worsened (eg, ensuring socioeconomically deprived communities have adequate access to green space). Most studies (n=71) that provided evidence for the effect of climate action on health inequalities seemed to focus on the effect of greener transport, blue and green infrastructure, and temperature changes on children, older populations, and those of a lower socioeconomic status (figure 3). The evidence was highly variable, with studies indicating positive, negative, and mixed effects. Actions grouped under buildings and infrastructure was the only domain with evidence as having exclusively positive effects on a range of characteristics, including age, ethnicity, socioeconomic, and spatial.

## Discussion

The Review identified extensive evidence on the effects of some types of climate interventions, predominantly in the domains of blue and green infrastructure, greener transport, and cleaner energy or reducing emissions, on health inequality. However, other studies found that the evidence currently available for some categories, such as biodiversity, business and industry, waste, and forestry, is insufficient.^26^^,^^64^ The scarcity of studies on the effects of nature-based solutions such as woodland creation or peatland restoration on health inequality is notable, as these are often touted to have co-benefits,^65^ although specific co-benefits on health and health inequality appear to be largely absent from the literature. Additionally, despite the large number of papers reviewed herein, there is an absence of focus, and therefore, an absence of coherent evidence across the literature, on the effect of climate mitigation or adaptation action(s) on health inequalities as a whole. This observation supports the findings of Luyten and colleagues, who highlighted an inadequate consideration of effects on health equality in climate adaptation and mitigation studies.^20^

An intervention’s context, or process of implementation, is consistently identified as important in influencing the nature or extent of its particular effects on health inequality.^13^^,^^66^ For example, interventions that promote increased active travel are more likely to reduce health inequalities when they include measures to remove the social, financial, and cultural barriers that exclude some groups from activities such as cycling.^67^^,^^68^ The distribution of green and blue infrastructure can also affect health inequalities either positively or negatively—for example, the need to both target communities most exposed to the health hazards of urban heat (often low-income or older residents) and protect these communities from the possible effects of gentrification (eg, physical and cultural displacement, rise in unaffordability) that can accompany neighbourhood improvements.^69^^,^^70^ These findings support public health approaches that suggest that population-level, upstream, or structural interventions (eg, universal public services, progressive taxation) are best suited for both improving outcomes overall and for reducing health inequalities.^71^^,^^72^ However, the typology of mitigation measures used to inform this Review did not include policies designed to tackle the vested economic interests of the fossil fuel and other harmful industries, the exclusion of which risks the growing health focus of climate action becoming mere healthwashing.^24^ The typology of measures was initially derived from The UK Government policy, suggesting insufficient political action in this influential area, with far-reaching consequences for health inequality.

The literature reviewed herein is highly interdisciplinary and methodologically varied, highlighting both the complexity and widespread relevance of this issue, and the inadequate alignment between various environmental science or public health science methods, or both, thus making comparable assessments of different interventions difficult. The absence of compelling, harmonised evidence on the health effects of mitigation has been noted elsewhere as a potential barrier to meaningful policy action.^17^ A large degree of complexity exists in the identified mechanisms, and for several categories of interventions, both positive and negative outcomes on health inequality are possible. These complex or overlapping mechanisms suggest that the mechanisms to health inequality outcomes are not linear, but more likely to be emergent from multiple, interacting co-impacts. Specific contexts and contingencies are, therefore, highly relevant and most likely to influence whether positive health co-benefits (or negative trade-offs) are triggered, or amplified, during the course of mitigation or adaptation action.^73^ The importance of context needs to be considered when seeking to identify which interventions might facilitate equigenesis, or improve health equality by design,^74^ and further research is needed on this topic. Despite challenges in precisely assessing the scale of these effects, Ürge-Vorsatz and colleagues argue that insights about the probable direction of co-impacts are valuable for informing decision making.^75^ These findings reinforce calls for greater use of systems approaches to assess these complex interactions more comprehensively.^76^

To the best of our knowledge, this systematic review is the first one designed to understand the effects of climate adaptation and mitigation measures in OECD high-income countries on health inequalities. The Review supports the widespread understanding that climate action is highly relevant for health outcomes, and adds insight to the open issue of how to best implement policies in ways that drive tangible health equality outcomes.^77^ The numerous complex mechanisms identified make these findings relevant for work exploring trade-off management in climate action, although this work exploring trade-off management in climate action is often focused on the environmental co-benefits and trade-offs.^78^^,^^79^ We argue here that health equality is a crucial, albeit underconsidered, outcome—one that other research has identified as necessary for facilitating a just transition.^36^^,^^66^^,^^80^ For example, the effects of different decarbonisation or electrification interventions on health inequality show the possible spatial injustice of air pollution reduction measures, which often lead to either a displacement of harm to different communities (including globally to low-income and middle-income countries) or generate health benefits that are predominantly felt in high-income (or otherwise, less disadvantaged) populations, or both.

The literature search also returned many studies on mitigation that considered the greening of the health-care or health-services sector (eg, green surgery, green anaesthesia, green radiology, more remote consultations, reduced waste). This decarbonisation activity is an important research area, given that the health-care system causes almost 5% of global greenhouse gas emissions.^81^ However, only one such study met the criteria for inclusion in this Review, by discussing the effects of climate change mitigation on health inequality in a clinical setting.^82^ The health community, as leading advocates of decarbonisation,^83^ has the potential to be influential in this area; therefore, we identify a need for stronger evidence on how the health-care system itself can contribute to both climate change mitigation and climate health justice.

Given the large number of studies that our initial search returned, we had to incorporate strict inclusion and exclusion criteria. However, many papers that studied OECD low-income examples and contexts were identified during the course of our research.^84^^,^^85^ Systematically assessing the effects of mitigation and adaptation approaches on health inequality remains a dire need in the context of low-income countries.^16^^,^^24^^,^^86^ In addition, climate actions undertaken by high-income countries sometimes have global health inequality implications that are experienced elsewhere (for example, global air pollution levels could increase or decrease depending on whether greenhouse gas emissions are fully removed or simply relocated); this area was not covered by this Review and needs further study. A further limitation of the paper is that, contrary to our original intention, a quality appraisal was not undertaken, given that only a few of the 158 included papers (n=29) could have been assessed this way (using Appraisal tool for Cross-Sectional Studies, Critical Appraisal Skills Programme [CASP] qualitative, CASP systematic review, and CASP cohort study checklists).^87^^,^^88^ As this Review focuses on the scientific literature in this field, a potential limitation could be that we have not included the evolving debates and evidence informing public polices and government action, which might be further captured in grey literature.

In conclusion, additional research is needed into the effects of some mitigation or adaptation actions (which have insufficient or no evidence) on health inequality, and we recommend that more studies build coherent health equality analysis into their evaluations. Such investigations are important not only for health inequality outcomes per se, but also because of the feedback loop identified between inequality and climate change, wherein each reinforces the other (illustrated by the example of air conditioning as an adaptation action).^4^ The heterogeneous nature of the included studies and methods also highlights some of the disparities between disciplinary practices and expertise. Tools developed to bridge some knowledge gaps, which climate, environmental, and public health scientists can all use, to make meaningful assessment of different interventions (involving a vast array of complex mechanisms) more straightforward, are the need of the hour for such an interdisciplinary topic area.^17^ For example, the use of social vulnerability indexes could be appropriate for research that spans both the climate and health sciences.^89^ A more comprehensive and systematic inclusion of health, and particularly health inequalities, in the broad field of climate study could, therefore, lead to insights that allow the scientific and policy communities to make faster and more effective progress towards reaching a just and sustainable world.

## Data sharing

All search strategies and typology documents will be made available online, courtesy of University of Glasgow.

## Declaration of interests

We declare no conflicts of interest.
