# Supplementary material for: The effect of climate mitigation and adaptation policies on health and health inequalities: a systematic review
Source: Lancet Planet Health. 2025 Jul 8;9(7):None. doi: 10.1016/j.lanplh.2025.06.001 (PMC12350827; doi:10.1016/j.lanplh.2025.06.001)
Supplement: Supplementary appendix 1 [file mmc1.pdf]

THE LANCET
Planetary Health

Supplementary appendix 1

This appendix formed part of the original submission and has been peer reviewed. We post it as supplied by the authors.

Supplement to: Hjelmskog A, Boyd J, Stevenson A, et al. The effect of climate mitigation and adaptation policies on health and health inequalities: a systematic review. *Lancet Planet Health* 2025. https://doi.org/10.1016/j.lanplh.2025.06.001

Medline (OVID): Searching from 1st January 2000-31st Dec 2023

Searches run on 24/03/2025

1	Climate Change/ or Global warming/	37102
2	(climate adj3 (extreme or resilience or induced or change* or impact* or disrupt* or hazard* or vulnerab* or function* or catastroph* or emergenc* or shift* or variab* or dynamic* or sensitiv* or mitigat* or adapt*)).ab,kf,ti.	93180
3	((global or planet or world or earth or worldwide or world-wide) adj3 (warming or heating)).ab,kf,ti.	17525
4	(climate adj3 (mitigat* or adapt*)).ab,kf,ti.	9751
5	(((((reduc* or sav* or tackl* or address* or halt* or stop* or abat* or remission* or moderat* or modif* or control* or zero or diminish* or decreas* or eliminat* or declin* or sink* sequester* or neutral or offset or storage or store or storing or captur* or perform* or efficien* or use* or usage or using or low or saving* or conserve* or adapt* or mal-adapt* or maladapt* or cope* or coping or climate-proof* or respond or responded or responding or response* or resilien* or safeguard* or sustain or prepar* or decarboni* or policy or policies or politics or polity or governance or govern or governing or tax* or emission trad* or pric* or tariff* or subsid* or regulat* or command) and control) or instrument* or incentiv* or standard* or offset* or plan* or information or label* or certific* or education or cooperat* or procurement* or investment* or credit* or bonds or bond or ban* or moratori* or loan or loans or monitoring or verification or campaign* or financ* or intervention*or instrument* or nudg* or Kyoto Protocol or Paris agreement or cap) and trade) or grant* or complian* or zoning or budget* or market* or participatory or public awareness or cooperation or cooperate or label* or guideline* or awareness or knowledge or guidance or grant scheme or advice* or skill* or fund* or protect* or restor* or maintain* or manage* or preserv* or scheme).ab,kf,ti.	7418878
6	4 and 5	5160
7	(biodivers* or diversif* or diverse or bisecur* or communicable disease or communicable or illness).ab,kf,ti.	895052
8	(forest* or woodland or tree* or creat* or plant*).ab,kf,ti.	1922960
9	(water or freshwater or water availab* or water qualit*).ab,kf,ti.	1114835

10	(agricult* or habitat or greenspace or green space* or green-space or green infrastructure or blue infrastructure or blue space* or greenbelt or urban green or green corridor or greenway or parkland).ab,kf,ti.	222982
11	(flood* or erosion or river* or coast* or stream).ab,kf,ti.	278678
12	(building* or infrastructur* or transport* or highway or rail* or aviation or energy sector or health care or social care or premise* or asset* or organization).ab,kf,ti.	1820431
13	(just transition or decarbonisation or transit or active travel* or active transport* or temperature or cold or hot or heat).ab,kf,ti.	1248465
14	(marine or ocean* or sea* or fjord or blue econom* or blue belt or natural carbon sink* or carbon sink or land or natur* or peatland or woodland or forest* or natural process* or hydrolog*).ab,kf,ti.	2904199
15	(business* or indust* or econom* or investment or green financ* or circular econom* or bio-economy or bioeconomy or green economy or resource efficiency or reuse or re-use or waste or zero-waste or green innovation or recycl*).ab,kf,ti.	1108405
16	((energy or fuel) adj3 (efficienc* or reduc* or renewable* or save or saving or clean*)).ab,kf,ti.	62823
17	((fossil fuel* or coal or oil or gas or carbon) adj3 (phas* out or exit* or transition* or transform or clos* or shift* or decommision* or shut* down or displac* or low* or net-zero or net zero or neutral)).ab,kf,ti.	29586
18	Public Health/ or Population health/	102480
19	Mental Health/	71972
20	"Quality of Life"/	302415
21	(wellbeing or well-being).ab,kf,ti.	178945
22	(health* or ill* or disease* or syndrom* or infect* or virus* or medical* or DALY or QALY or life year or life-year or burden of disease or mortalit* or morbidit* or survival* or death* or hospital* or emergenc* or prevalen* or incidenc*).ab,kf,ti.	15011617
23	(lifestyle or life-style or health behaviour or health behavior or diet or physical activ*).ab,kf,ti.	722444
24	Socioeconomic Factors/ or Social welfare/	187156
25	(injustic or discriminat* or inequalit* or disparit* or equit* or inequit* or equalit* or socioeconomic or socio-economic or welfare or social determinant* or social class* or social grade or sociodemographic* or social gradient* or disadvantage* or unemploy* or underemploy* or poverty or impoverished or low	2481683

	income or low-income or occupation* or education* or class or income).ab,kf,ti.	
26	(gender or sex or sexual or ethnic* or race or racial or caste or disability or disabled or age or old* or young*).ab,kf,ti.	5695331
27	1 or 2 or 3	110422
28	6 or 7 or 8 or 9 or 10 or 11 or 12 or 13 or 14 or 15 or 16 or 17	8778238
29	18 or 19 or 20 or 21 or 22 or 23	15401595
30	24 or 25 or 26	7430171
31	29 or 30	17975843
32	27 and 28 and 31	40636
33	limit 32 to yr="2000-2023"	31877
34	exp animals/ not humans.sh.	5320041
35	33 not 34	25478
36	Limit 35 to dt = 20220924-20231231	5224

Scopus: Searching from 1st January 2000-31st Dec 2023

1	(TITLE-ABS-KEY (climate AND change OR global AND warming))	101,971
2	TITLE-ABS-KEY (climate PRE/3 (extreme OR resilience OR induced OR change* OR adapt* OR mitigat*))	565,378
3	TITLE-ABS-KEY (global OR planet OR world OR earth OR worldwide OR world-wide PRE/3 (warming OR heating))	133,676
4	TITLE-ABS-KEY(climate PRE/3 mitigat* OR adapt*)	47,439
5	TITLE-ABS-KEY (net-zero OR reduc* or sav* or tackl* or address* or halt* or stop* or abat* or remission* or moderat* or modif* or control* or zero or diminish* or decreas* or eliminat* or declin* or sink* OR sequester* or neutral or offset or storage or store or storing or captur* or perform* or efficien* or use* or usage or using or low or saving* or conserve* or adapt* or mal-adapt* or maladapt* or cope* or coping or climate-proof* or respond or responded or responding or response* or resilien* or safeguard* or sustain or prepar* or decarboni* or policy or policies or politics or polity or governance or govern or governing or tax* or emission* or pric* or tariff* or subsid* or regulat* or instrument* or incentiv* or standard* or offset* or plan* or information or label* or certific* or education or cooperat* or procurement* or investment* or credit* or bonds or bond or ban* or moratori* or loan or loans or monitoring or verification or campaign* or financ* or intervention*or instrument* or nudg* or Kyoto Protocol or Paris agreement or grant* or complian* or zoning or budget* or market* or participatory or cooperation or cooperate or label* or guideline* or awareness or knowledge or guidance or grant scheme or advice* or skill* or fund* or protect* or restor* or maintain* or manage* or preserv* or scheme)	5,669
6	TITLE-ABS-KEY (health* OR ill* OR disease* OR syndrome* OR infect* OR virus* OR medical* OR daly OR qaly OR life AND year OR life-year OR mortality OR morbidit* OR survival* OR death* OR hospital* OR emergenc* OR doctor* OR gp OR prevalen* OR incidenc* OR lifestyle OR life-style OR behaviour OR behavior OR co-benefit*)	11,094,448
7	TITLE-ABS-KEY (injustic OR discriminat* OR inequalit* OR disparit* OR equit* OR inequit* OR equalit* OR socioeconomic* OR socio-economic* OR social AND determinant* OR social AND class* OR social AND grade* OR sociodemographic* OR social AND gradient* OR disadvantage* OR unemploy* OR underemploy* OR poverty OR impoverished OR low-income OR low AND income OR occupation* OR education* OR class OR income OR gender OR sex OR ethnic* OR race OR racial OR caste OR disability OR disabled OR age OR old* OR young*)	269,269

8	1 OR 2 OR 3	662,281
9	4 OR 5	52,915
10	6 OR 7	11,230,100
11	8 AND 9 AND 10	4,070
12	Limit year of publication to 2000-2023.	3,223
13	Limit year of publication to 2022-2023	1,010

Web of Science Core Collection (Database): Searching from 1st January 2000-31st Dec 2023

1	((TI=(climat* NEAR/3 change* OR changing OR impact* OR disrupt* OR hazard* OR vulnerab* OR function* OR extreme* OR catastroph* OR emergenc* OR shift* OR variab* OR dynamic* OR sensitiv*)) OR TI=(global OR plant OR world OR earth OR worldwide OR world-wide NEAR/3 warming OR heating)) OR TI=(climate change OR global warming)	8,606,694
2	AB=(climat* NEAR/3 change* OR changing OR impact* OR disrupt* OR hazard* OR vulnerab* OR function* OR extreme* OR catastroph* OR emergenc* OR shift* OR variab* OR dynamic* OR sensitiv*) OR AB=(global OR plant OR world OR earth OR worldwide OR world-wide NEAR/3 warming OR heating) OR AB=(climate change OR global warming)	27,998,254
3	TS=(climate NEAR/3 (mitigat* or adapt*))	63,377
4	(TS=(climate mitigat* OR climate adapt* OR reduc* OR tackl* OR address OR halt OR stop OR mal-adapt OR maladapt OR respond OR safeguard OR sustain OR prepar*)) OR TS=(policy OR policies OR politics OR governance OR govern OR governing OR regulat* OR subsid* OR incentiv* OR Kyoto Protocol OR Paris agreement OR public awareness OR guideline* OR campaign OR awareness OR knowledge OR guidance OR grant scheme OR education OR label* OR plan* OR advice OR skill* OR fund* OR protect* OR restor* OR maintain* OR conserv* OR manage* OR scheme*)	32,665,219
5	TS=(biodivers* OR diversif* OR diverse OR biosecur* OR communicable disease OR communicable OR illness OR forest* OR woodland OR tree* OR creat* OR plant* OR water OR freshwater OR water availab* OR water qualit* OR agricult* OR land use OR land-use OR agri-environment OR habitat* OR greenspace OR green-space OR green infrastructure OR blue infrastructure OR blue space* OR greenbelt OR urban green OR green corridor OR greenway OR parkland OR flood* OR erosion OR river* OR coast* OR stream OR building* OR infrastructur* OR transport OR highway* OR rail* OR aviation OR energy sector OR health care OR health-care OR social-care OR social care OR premise*	27,680,156

	OR asset* OR organisation OR just transition OR decarbonisation OR transport* OR transit OR active travel* OR active transport* OR temperature OR cold OR heat OR hot OR marine OR ocean* OR sea* OR fjord OR blue econom* OR blue belt OR natural carbon sink* OR land OR natur* OR peatland OR woodland OR forest* OR habitat OR natural process* OR hydrolog* OR business* OR indust OR econom* OR investment OR green financ* OR circular econom* OR bio-economy OR bioeconomy OR green economy OR resource efficiency OR reuse OR re-use OR waste OR zero-waste OR green innovation OR recycl*)	
6	(TS=(energy OR fuel NEAR/3 efficen* OR reduc* OR renewable* OR save OR saving OR clean*)) OR TS=(fossil fuel* OR coal OR oil OR gas OR carbon NEAR/3 phas* out OR exit* OR transition* OR transform OR clos* OR shift* OR decommision* OR shut* down OR displac* OR low* OR net-zero OR net zero OR neutral*)	28,314,168
7	(TS=(health* OR ill* OR disease* OR syndrome* OR infect* OR virus* OR medical* OR DALY OR QALY OR life year OR life-year OR burden of disease OR mortality OR morbidit* OR survival* OR death* OR hospital* OR emergenc* OR doctor* OR GP OR general practitioner OR prevalen* OR incidenc* or co-benefit*)) OR TS=(lifestyle OR life-style OR health behaviour OR health behavior OR diet OR physical activ*)	20,867,925
8	TS=(injustic OR discriminat* OR inequalit* OR disparit* OR equit* OR inequit* OR equalit* OR socioeconomic* OR socio-economic* OR social determinant* OR social class* OR social grade* OR sociodemographic* OR social gradient* OR disadvantage* OR unemploy* OR underemploy* OR poverty OR impoverished OR low-income OR low income OR occupation* OR education* OR class OR income)	6,294,354
9	(TS=(gender OR sex OR ethnic* OR race OR racial OR caste OR disability OR disabled OR age OR old* OR young*))	8,774,135
10	#4 OR #5 OR #6	51,900,900
11	#3 AND #10	63,377
12	#1 OR #2	31,364,264
13	#7 OR #8 OR #9	28,127,315
15	#11 AND #12 AND #13 and	24,994
16	Limit to 2000-2023 publications.	20,342

17	Limit to publication date between 20220924- 20231231	**3,745**
