# Supplementary material for: The effect of climate mitigation and adaptation policies on health and health inequalities: a systematic review
Source: Lancet Planet Health. 2025 Jul 8;9(7):None. doi: 10.1016/j.lanplh.2025.06.001 (PMC12350827; doi:10.1016/j.lanplh.2025.06.001)
Supplement: Supplementary appendix 2 [file mmc2.pdf]

THE LANCET
Planetary Health

Supplementary appendix 2

This appendix formed part of the original submission and has been peer reviewed.
We post it as supplied by the authors.

Supplement to: Hjelmskog A, Boyd J, Stevenson A, et al. The effect of climate mitigation and adaptation policies on health and health inequalities: a systematic review. *Lancet Planet Health* 2025. https://doi.org/10.1016/j.lanplh.2025.06.001

**Table 3 note**	
A	Adaptation
M	Mitigation
+	Positive impact on health/health inequality is reduced
-	Negative impact on health/health inequality is increased
~	No effect on health/health inequality is observed
CVD	Cardiovascular Disease
COPD	Chronic Obstructive Pulmonary Disease
DALY	Disability Adjusted Life Years
GHG	Greenhouse Gas
SES	Socioeconomic Status
YLL	Years of Life Lost

Table 3a: Original research papers

**Author, Year & Country**	**Study Design**	**Climate Action**	**Mitigation (M) and/or Adaptation (A)**	**Health Outcome**	**Inequality Measure**	**Impact on Health**	**Impact on Health Inequality**
Achebak et al., (2023), Spain (1)	Longitudinal	Air conditioning and heating	A	Heat and cold-related mortality	Age, Income, Spatial, Education,	(+)	(-) Heat-related mortality risks were positively associated with average retirement income. Ageing also attenuated the decrease in cold-related mortality. The warmest regions

							had the highest prevalence of houses with AC, whereas the coldest regions had the highest prevalence of houses with central heating. Reducing this spatial inequality could improve health outcomes from elderly populations.
Africa et al., (2019), Global (2)	Theoretical	Biophyllic Design Practices	M	Mental fatigue, psychological stability & sleep-wake cycles	SES	(+)	(-) more accessible to less deprived populations
Anderson et al., (2018), USA (3)	Policy evaluation	Cap-and-trade mitigation programme	M	Mortality, asthma, strokes, heart attacks, & lung disease	SES & Spatial	(+ ~)	(+ ~) Sources of pollution (both mobile and stationary) are disproportionately present in disadvantaged communities, so reductions in these can offer health benefits to communities that have most to gain (+) However, the Cap-and-Trade program tackles stationary sources of pollution, which are only responsible for a minority of pollutants found in disadvantaged communities (~) Use of offsets (which may signal missed opportunities to

							reduce levels of co-pollutants) are found to be independent of a site's location in a disadvantaged or non-disadvantaged communities (~)
Anderson et al., (2021), Canada (4)	Health Equity Impact Assessment	Green Infrastructure	M & A	Community mental & physical wellbeing	Age, Income, Housing Tenure & Disability	(+)	(+) Greenspace benefits have more significant impact for disadvantaged groups
Angradi et al., (2022), USA (5)	Cross-sectional	Natural Capital	M & A	Human well-being	Area Deprivation Index & Social Vulnerability Index	(+ -)	(+) The effect of walkability on health was much higher in low-income census tracts than in more affluent tracts - suggesting that walkability is less relevant to the wellbeing of those that are more affluent.
Appolloni et al., (2020), Italy (6)	Standards Proposal	New Building Regulations	M & A	Health & wellbeing generally	SES	(+)	(+) More energy efficient homes mean that socioeconomically disadvantaged people can meet the energy costs of their house, improving health and wellbeing
Arai et al., (2020),	Cross-sectional	Non-use of air conditioners	M	Wellbeing & mortality risk	Sex & Age	(-)	(-) Non-use of air conditioners increases heat-related stroke

Japan (7)							risk for the elderly
Asikainen et al., (2017), Finland (8)	Modelling Study	Greenhouse Gas Abatement	M	All-cause mortality, restricted activity days, chronic bronchitis, cardiovascular & respiratory hospital admission	Age & Income	(~)	(~) Older people and people with lower income continue to experience higher exposure to air pollutants
Astell-Burt et al., (2020), Australia (9)	Cohort	Urban Green Space	M & A	Anti-dementia medication prescription & dementia detected at hospitalisation or death	Area-level socio-economic disadvantage	(+ -)	(~) Socioeconomic disadvantage fully attenuated associations between green space and anti-dementia medication records
Bailey et al., (2019), Greece (10)	Health Impact Assessment	Interventions for reducing the pollution from wood burning activities	M	Preventable average daily mortality	SES	(+)	(+) Reduced number of deaths for all groups, but particularly for low SES
Baldwin et al., (2020), Australia	Qualitative	Urban Greening in aged-care facilities	M & A	Health & wellbeing generally	Age, Sex & Ethnicity	NA	(+) Appropriate, culturally-sensitive greenspace has beneficial health effects on

(11)							target population
Bell et al., (2019), Europe (12)	Report	Green spaces, energy efficient housing, active transport and sustainable diets	M & A	Physical activity, healthy eating & mental wellbeing	Age, Sex, Household Income, Disability & Ethnicity	(+ - ~)	(+ -) Younger and older females less likely to use paths/green spaces for active travel and physical activity and poorer households used path more frequently than richer households. Children were most likely to use parks for physical activity and people from different ethnic background co-used park and facilities. Urban cycling is most frequent among middle-aged males that are highly educated and employed.
Bikomeye et al., (2021a), USA (13)	Conceptual Framework	Urban Green Space	M & A	Mental health & physical activity	Age, Ethnicity, Low income & urban neighbourhoods	(+)	(+) if targeted at in schools and low-income neighbourhoods
Braubach et al., (2015), Europe	Modelling study	Local transport interventions to increase public transport and electric vehicle	M	Wellbeing impacts from traffic noise	Income	~	~

(14)		use and reduces private road traffic					
Buchin et al., (2016), Germany & USA (15)	Evaluation modelling study	Countermeasures to urban heat islands (e.g., cool pavements, green roofs, air conditioning)	A	Heat-related mortality	Only looks at those aged 65+	NA	(+) Reduction in ambient temperatures reduces mortality in 65+ population
Burke et al., (2020), USA (16)	Modelling study	Fuel Management Interventions to reduce wildfires	M & A	Premature deaths avoided per year	Only looks at those aged 65+	NA	(+) Deaths in 65+ population avoided by reducing exposure to PM2.5
Burlotos et al., (2023), USA (17)	Retrospective Case Analysis	Heatwave response: cooling centers, public health messaging, wellness checks and outreach approaches.	A	Heat-related mortality	Age, gender, ethnicity & housing status	(+)	(+) As a result of outreach efforts only 4 of 72 deaths that did occur were those experiencing homelessness. The majority of deaths that occured were for males (67%), over 60 years old (78%) and of white race and non-hispanic ethnicity (82%).
Carlton et al., (2019), USA (18)	Cross-sectional	Home ventilation	A	Respiratory symptoms	Low-income population	NA	(-) Increasing ventilation in low-income urban neighbourhood increases exposure to outdoor pollutants and worsens health outcomes for the low-income,

							multi-ethnic sample population
Chakraborty et al., (2022), USA (19)	Longitudinal	Afforestation	M & A	Heat-related mortality and morbidity	Income	(+)	(+) Lower income populations live in regions with higher Surface Urban Heat Island (SUHI) intensity and a lower percentage of tree cover (14.8% compared to 20.7% in high income areas).
Chen et al., (2014), Australia (20)	Simulation Modelling	Urban vegetation to shade buildings and cool ambient air temperature	A	Heat-related mortality	Sex	(+)	(~) Equally positive impacts for both males and females
Chiabai et al., (2018), UK (21)	Conceptual framework	Green spaces	M & A	Mental health, neurological disease, cardiovascular disease, respiratory disease, digestive disease & musculoskeletal disease	Age & SES	(+)	(~) Noted that social, economic and demographic characteristics not only influence health, but also affect the way in which green space interacts with it. Special attention should be paid to vulnerable socioeconomic groups, ageing populations and poorer groups.
Cifuentes et al.,	Modelling study	Reducing Greenhouse Gas	M	Mortality, infant mortality, chronic	Age (children)	(+)	(+) effects for children as well as adults

(2001), USA & Mexico (22)		emissions in energy, transport, residential and industrial sectors		bronchitis & person days of work loss or other restricted activity			
De'Donato et al., (2018), Italy (23)	Cohort	Heat Prevention Plan	A	Mortality from all-natural causes and heat-related causes	Population aged 65+	NA	(+) for extreme temperatures only
Dietz & Pryor (2022), USA (24)	Report	Sustainable diets & active transport	M	Health generally	Marginalised communities	(+)	(+) reduced motorised transport reduces exposure to poor air quality for marginalised communities
Dufty (2022), Australia (25)	Report	Heat Refuges	A	Heat exhaustion, heat syncope, heat stroke, death	Age, gender, SES, people not fluent in English, households without a car, single-parent families, single & older people (65+), pre-existing illness, disability	(+)	(+ -) People of lower socio-economic status are less likely to have air-conditioning within the home. As such, public refuges during heatwaves are of greater importance to this group.<br>Certain groups are at greater physiological risk from the negative impacts of heatwaves i.e. children and older people, women, those with pre-existing

					status, medication/drug use, people experiencing homelessness, employment, Indigenous peoples, people from culturally and linguistically diverse (CALD) backgrounds		conditions or struggling with drug addiction. As such, the presence of public refuges is more likely to benefit them during heatwaves.<br>However, access to these refuges is likely to depend on other factors: children of single parents are less likely to be able to make it to refuges if their parent is working; people without cars may struggle to access refuges.
Farchi et al., (2017), Italy (26)	Meta-analysis	Reduced meat consumption	M	Avoidable deaths from colorectal cancer, & cardiovascular disease; years of life gained; changes to life expectancy	Geographical area, sex & age	(+)	(+) could reduce inequalities in mortality risk for cardiovascular and colorectal cancer between men and women; as men eat more red meat and are at higher risk
Ford et al., (2018), Canada (27)	Case study	Community-based adaptation programmes	A	Health generally	Indigenous population	NA	(+) Tailoring adaptation to the needs and characteristics of indigenous population improves health and wellbeing and avoids unintentional negative

							impacts
Fraser & Chester, (2017a), USA (28)	Case studies	Increase public transportation use	M	Extreme temperature-related mortality, heat cramps, heat stroke and heat exhaustion & exacerbation of pre-existing chronic conditions such as respiratory & cardiovascular diseases contributing to emergency room visits, hospitalizations, & premature death.	Residential density & those living in areas with poor walkability features. Paper also notes that the elderly, young children, those living in poverty, and those with underlying medical conditions are predisposed to heat-related morbidity and mortality	(-)	(-) Use of public transport increases exposure to air pollution and extreme temperatures (through waiting times, walking to transit stops), causing more harm to vulnerable groups
Fraser & Chester, (2017b), USA (29)	Case studies	Public transportation: use and frequency of transit	M & A	Health outcomes for transit riders during periods of extreme heat	Riders more vulnerable to heat (implies the elderly)	(+)	(+) Optimisation of route frequencies to meet the needs of vulnerable populations reduces their time spent exposed to hazards and minimises health harms

Fratini (2023), Italy (30)	Natural Experiment/ Mixed Methods	Microforests	M & A	Wellbeing	Age (paper focuses on children)	(+)	(+) Microforests can connect children with nature, instilling values which encourage them to care for it as they grow; they can also serve as sites to teach and prepare children for changes to be expected from climate change, thereby developing their adaptive capacity from a young age.
Ferrari et al., (2020), Italy (31)	Cross-sectional	Diets that minimise Greenhouse Gas emissions	M	Nutritional completeness of diets	Sex	(+)	(-) iron requirements cannot be met for women
Fitzroy et al., (2012), Global (32)	Policy Review	Retrofitting buildings, investing in green energy and greener transport.	M	Happiness & subjective wellbeing	Income & rurality	(+)	(+) Quality of life improves for traditionally marginalised (rural and disadvantaged) communities who may typically be unable to afford access to public transport
Friel et al., (2011), Global (33)	Theoretical	Policies and programs in urban planning and design, workplace health and safety, and urban	M & A	Heat-related health risks, access to & consumption of healthy foods	Income, employment, land ownership, age, sex & race	(+)	(+-~) Noted that these measures need to target vulnerable groups (e.g., low income, unemployed, elderly etc) that are at greater risk

		agriculture					
Fujimoto et al., (2023), Japan (34)	Cross-sectional	Heat adaptation strategies: drinking water, air conditioning and not living alone		Heat Stroke	Age (paper focuses on people aged 65+)	(+)	(+) Older people have more difficulty adapting to warm environments, so are more likely to develop heatstroke in the absence of adaptation measures. Explanatory variables of heatstroke episodes included: male sex, living alone and having an underlying medical condition. Difficulty drinking water and the absence of an air conditioner were also found to be significantly related to heatstroke-related ambulance transports.<br>According to the modelling results, reducing the number of older people living alone over time would result in an up to 15% decrease in the number of heatstroke-related transports. Averting all 3 risks (living alone, difficulty drinking water and the absence of an air conditioner) would result in a 20% decrease

							in heatstroke by decreasing the proportions with risk factors at 30%.
Gabbe et al., (2023), USA (35)	Cross-sectional	Cooling Centres	A	Heat Exposure	Housing Status	(+)	(+ -) Cooling centres if accessed by those experiencing homelessness would reduce the risk of heat exposure and as a result dehydration, sunburn and heatstroke. People found that the cooling centres were neither well-advertised nor conveniently located. There was an element of digital exclusion whereby unhoused people were less likely to have access to internet/devices/power sources from which they could find information on the cooling centres. Policies at cooling centres were also exclusionary to some unhoused people: there were restrictions on curfews, limited hours, policies against intoxication or substance use, policies against

							pets, and lack of storage options for personal belongings. Several participants favoured using public libraries during heat periods instead of cooling centres. One participant reported experiencing less stigma at a public library.
Gallagher & Holloway (2022), USA (36)	Modelling study	Decarbonisation: carbon-free electricity, carbon-free industrial activity and carbon-free light duty vehicle transportation	M	Pollutant Exposure; proxy for respiratory health, cardiovascular diseases	Ethnicity	(+)	(+) All decarbonisation policies lead to reductions in ambient PM2.5 concentrations. Specifically, decarbonising light duty vehicles is more beneficial for Hispanic populations and communities of color than decarbonising industry.
Garcia (2023), USA (37)	Cross-sectional	Zero-emissions vehicles (ZEV)	M	Rates of asthma-related emergency departments visits.	Educational Attainment (proxy for SES)	(+)	(-) Lower rate of ZEV adoption in more socioeconomically deprived neighbourhoods
Giles-Corti et al., (2010), Europe,	Policy analysis	Active transport	M	Mortality, general fitness levels, obesity levels, respiratory	Age & Sex	(+)	(+-)<br>(-) Girls benefit more than boys from increased cycling<br>(+) Larger health gains may be

North America & Australia (38)				conditions, general improvements in physical & mental health			felt in urban populations who are more exposed to harmful roadside pollutants and congestion
Gölitzer et al., (2023), Germany (39)	Cross-sectional	Taking stairs instead of elevators	M	Physical activity (taking the stairs)	Sex	(+)	(+) Equalising effect: women increasingly likely to take the stairs after the intervention, narrowing the pre-existing gap between women and men
Gong et al., (2022), England, UK (40)	Modelling study	Using fans as cooling strategy	A	Heat-related dementia hospital admissions	Age, deprivation, pre-existing condition (people living with dementia).	(-)	(-) Vulnerable group be more inclined than other groups to utilise fans as a cooling strategy but these may in fact exacerbate the risk of higher temperatures leading to heat-related hospital admissions.
Green et al., (2013), USA (41)	Health Impact Assessment	Climate Smart Policies (e.g., active travel, 20-min neighbourhoods, carbon taxes, individual & employer incentives e.g.,	M	Chronic disease; heart disease, stroke, diabetes, cancer, obesity & all-cause mortality	Age, Disability, Ethnicity & Income	(+)	(+) Prioritizing investments and thoughtful implementation of active transportation policies and programs in vulnerable communities could improve inequitable health outcomes for vulnerable populations (e.g. those disadvantaged by disability or ethnicity) in the

		car sharing)					Portland metropolitan region
Grummon et al., (2023), USA (42)	Modelling study	Dietary change from higher- to low-carbon foods	M	Dietary quality assessed using the Healthy Eating Index	Age, Gender, Race/Ethnicity	(+)	(+~) Variation observed between different categories of substitution and dietary improvements of different groups
de Guzman et al., (2022), USA (43)	Mixed Methods	Tree Stewardship programme	A	Heat health action index	Income, Ethnicity	(+)	(+) Increasing tree coverage and albedo of roofs and pavements in urban area (LA) can reduce heat-related mortality by upwards of 25%, especially in low-income communities and communities of color
Hanus et al., (2019), USA (44)	Modelling study	Solar photovoltaic in educational institutions	M	Healthcare costs	At risk populations; elderly, asthmatics & low-income families	(+)	(+) Improved air quality reduces need for health treatment of respiratory conditions and CVD in vulnerable populations
Hebbern et al., (2023), Canada (45)	Modelling study	GHG emission reduction scenarios under various SSPs (Shared	M	Mortality	Age	(+)	(~) Mortality increases least under the high mitigation scenario, but elderly population (over 65s) experiences the highest gains in mortality under

		Socioeconomic Pathways)					all scenarios
Heudorf & Schade (2014), Germany (46)	Cross-sectional	Heat-health action plans and warning systems	A	Mortality	Age	(+)	(+) Although HHAPs mitigate excess mortality associated with heat waves across all age groups, there was still a significant increase in excess mortality during the heatwave in 2010 for those aged 80 and above
Hochard et al., (2022), USA (47)	Natural experiment	Hurricane Forecast Early Warning System	A	Adverse Birth Outcomes	Sex, Age (pregnant women and infants), SES	(-)	(-) Hurricane forecasting led to unnecessary cancellation of routine care and medical appointments, causing more indirect harm than the direct effects of the storm (Maladaptation)
Hoffman et al., (2021), Germany (48)	Cross-sectional	Radiant cooling systems in hospital patient rooms	A	Length of Hospital Stay, COPD Assessment Test (CAT), & Body Weight	Age	(+)	(+) could be more beneficial for older people that are at greater risk
Izquierdo et al.,	Health Impact	Air Quality & Climate Change	M	Mortality	Sex	(+)	(~) no difference in mortality rates results between men and

(2020), Spain (49)	Assessment	Plan for Madrid City					women
Jee et al., (2023), Global (50)	Commentary	Fossil Fuel divestment/Renewable energy investment by paediatricians	M	allergic rhinitis and asthma; heat-related illnesses; premature birth; injuries from severe storms and fires; vector-borne diseases; and mental illnesses.	Age (Focus on children) plus references to race and immigration status.	(+)	(+) Reducing/removing fossil fuels' contribution to climate change will reduce the exacerbated health impacts of climate change that are disproportionately felt by vulnerable populations (particularly children)
Johnson et al., (2020), USA (51)	Modelling study	Buildings: City legislation and rulemaking, technical support and financing & Transportation: Federal policies (Tier 3 vehicle emissions and fuel standards, heavy duty vehicle GHG standards, renewable fuel	M	Premature deaths, hospitalisations, emergency department visits for respiratory & cardiovascular disease	Neighbourhood Income	(+)	(+) Almost 50% more avoided premature deaths in the poorest versus the wealthiest neighbourhoods.<br><br>Greatest reductions in avoided asthma emergency department visits in low-income neighbourhoods compared to the wealthiest neighbourhoods (for similar declines in ambient PM2.5), although the number of emergency department visits for low-income neighbourhoods

		standard) State inspection and maintenance programs, City incentives for light- and heavy-duty private fleet (parking rules, access restrictions) and city purchasing policy for city fleet & City/state legislation and rulemaking inter-state authority policy					remain significantly higher than for wealthy neighbourhoods in all scenarios.
Johnson et al., (2022), New Zealand (52)	Cross-sectional	Applying indigenous knowledge to adapt to food insecurity	A	Stress & access to fresh food	Age, indigenous groups, location & employment	(+)	(+) Wellbeing benefits experienced by Maori women
Johnson et al., (2023), New Zealand	Qualitative (cross-sectional)	Water storage project	A	Asthma, dehydration, skin cancer, food and energy insecurity	Indigeneity, gender, class, race, single-parenthood	(-)	(-) Low-income Maori women most likely to be impacted by the negative health effects of the intervention

(53)							
Kabisch et al., (2016), Europe (54)	Cross-sectional	Nature-based solutions	M & A	Rates of respiratory diseases & obesity	Income	(+)	(-) Gentrification effects exclude lower income residents (who would have most to gain) from the improved, more attractive neighbourhoods
Karakas et al., (2023), England and Wales, UK (55)	Health Impact Assessment	School building retrofit	M	Asthma cases	Age (paper focuses on children)	(~)	(+) Small net decrease in asthma cases experienced by target population (children)
Kim et al., (2023b), South Korea (56)	Case study: Socio-spatial analysis	Urban cooling, including urban parks and forests, cooling centres	M & A	Heat Stress	Age, Economic	(+)	(-) Neighbourhoods with highest proportions of elderly residents and with lowest land prices have least access to cooling resources
Kingsborough et al., (2017), UK (57)	Modelling study	Green infrastructure & air conditioning	M & A	Heat-related deaths	Income	(+ -)	(-) those that use air conditioning tend to be wealthy
Klopfer & Pfeiffer (2023), Germany	Case Study spatial analysis	Green Infrastructure	M & A	Heat Exposure	Social Deprivation, 'non German' used as proxy for	(+)	(-) Green space inequalities are experienced along existing socioeconomic lines

(58)					disadvantage		
Konijnendijk (2022), Spain (59)	Theoretical	Urban greenspace	M & A	Mental health, wellbeing, & all-cause mortality	SES	(+)	(+) Noted that green space needs to be targeted in more deprived areas to reduce proximity barriers currently faced by more deprived communities
Kubes & Hayes (2019), USA (60)	Modelling study	Energy Efficiency in buildings and Electric Vehicles	M & A	Morbidity & mortality	Spatial & geographical	(+ -)	(+ ~) some geographic locations identified as having most to gain
Kuchcik et al., (2016), Poland (61)	Case study	Tree & shrub planting in urban areas	M & A	Allergies	Age	(-)	(-) greater impacts for younger people/children
Lane et al., (2023), USA (62)	Programme Evaluation	Air Conditioning	A	Heat exposure, feeling sick	Age, SES, Ethnicity (program targeted older, low-income residents, predominantly people of color)	(+)	(+) The program helped participants stay home safely during hot weather (low-income residents and primarily people of color)
Long et al., (2023),	Modelling study	End-of-pipe technologies and	M	Premature deaths, work hours lost,	Spatial	(+)	(+~) This study predicted climate actions would enable western

Japan (63)		Electrification		economic losses, asthma cases, bronchodilator usage			Japan to benefit from PM2.5 control measures, whereas the entire country would benefit from ozone pollution reduction
Lowe et al., (2016), Europe (64)	Modelling study	Heat-Health Action Plans	A	Mortality	Age & SES	(+)	Not explicitly modelled but noted that more vulnerable populations are more likely to be included in general mortality rates
Lucas & Pangbourne (2014), Scotland, UK (65)	Evaluation framework	Transport policy for climate mitigation (e.g., reducing cost of public transport, cycling infrastructure)	M	Accidents & physical activity	Income, Spatial, Age, Sex, Ethnicity & Disability	(+ - ~)	(-) Cycling has barriers of accessibility, affordability, and cultural awareness that disadvantage low-income groups, disabled people, and ethnic minorities, meaning that high-income, white, non-disabled populations experience the majority of health benefits from physical activity<br><br>(~) Improvements to air quality and road safety will benefit all
Luo et al., (2022),	Modelling framework	Decarbonising power systems	M	Premature mortality	SES & Ethnicity	(+)	(-) None of examined decarbonization strategies

USA (66)							mitigate the disparity which means that black and lower-income populations receive disproportionately higher air pollution damages. Instead, most strategies considered are found to exacerbate this inequality.
Mailloux et al., (2022), USA (67)	Modelling study	Eliminating energy related emissions from electricity fuel use, residential/commercial fuel use, industrial fuel use, on-road vehicles, non-road vehicles and oil & gas production and refining	M	Morbidity & mortality	Spatial	(+)	(+) Nationwide action benefits all regions
Marí-Dell'Olmo et al., (2022), Spain, USA, Chile,	Conceptual Framework	Active travel, Energy efficiency in buildings, More sustainable diets, Reducing food waste,	M & A	General Health	Vulnerability	(+)	(-) Existing social inequalities have a negative impact on the adaptive capacities of vulnerable groups. Adaptation strategies should not only include, but prioritise,

South Africa, Mexico, Australia (68)		Surveillance and warning plans					the most vulnerable e.g. heat wave action plans should take special consideration for vulnerable groups, such as elderly people living alone (especially women)
Marvuglia et al., (2020), Europe (69)	Microsimulation model	Green roofs	M & A	Mortality	Age 65+	NA	(+) Health benefits for 65+ age group
McMichael & Kovats (2000), The Netherlands (70)	Theoretical	Public Health Responses: Health Early Warning Systems, Public Education, Improved healthcare facilities, vaccinations, & environmental management (e.g., sea walls, housing)	M & A	Health generally, heat stress, vector-borne diseases & infectious diseases	Spatial	(+)	(~) longer term improvements in social and material conditions of life are required to reduce health inequalities
Meyerricks & White	Ethnographi	Community climate action	M	Wellbeing	SES & Spatial	(+)	(+~) Population in question experiences wellbeing benefits,

(2021), Scotland, UK (71)	c	projects to reduce local carbon emissions					but the paper notes that small-scale community initiatives are unlikely to tackle the root causes of existing inequalities
O'Neill et al., (2018), USA (72)	Modelling study	Reduced emissions scenarios	M	Vector-borne diseases, heat-related health & mortality risk	SES	(+)	Not considered but noted that if interventions are targeted at low-income households this may reduce inequalities
Oka et al., (2023), Japan (73)	Modelling Study	Reducing Greenhouse Gas Emissions (under Shared Socioeconomic Pathways Scenarios (SSPs)) and general 'Heat Adaptation', either physiological or non-physiological	M & A	Heatstroke Incidence and Number of Patients with Heatstroke Transported by Ambulance (NPHTA)	Age	(+)	(~) The vulnerable populations (oldest and youngest groups) experience the smallest proportional gains in health outcomes, therefore health inequalities do not narrow.
Ortiz et al., (2023), USA (74)	Modelling study	Decarbonising the energy sector. Transition from fossil fuel power to renewable and	M	Avoided deaths, hospitalizations, and loss of days worked	Social Vulnerability Index (SOVI)	(+)	(+) The benefits of avoided mortality are highest for those counties in the top quintile of poverty (income below 150% of the poverty line)

		nuclear energy					
Patterson et al., (2021), Sweden (75)	Health Impact Modelling	Sustainable diets	M	Year of Life Lost, Ischaemic Heart Disease, Ischaemic stroke, type 2 diabetes, & colorectal cancer	Sex	(+)	(-) greater positive impacts for men
Perera et al., (2020), USA (76)	Modelling study	USA's Regional Greenhouse Gas Initiative (market-based programme to reduce emissions from the electric power sector)	M	Pre-term birth, low birthweight, autism & asthma	Age: focuses on children only & Spatial	NA	(+) note that size of health effects varies according to geography
Perez et al., (2015), Switzerland (77)	Modelling study	Transport-related Greenhouse gas reduction policies (e.g., switching to electric vehicles, active travel)	M	Mortality, Morbidity & DALYs	NA	(+)	Not considered explicitly but noted that socioeconomic status should be taken into consideration
Peters et al., (2020), USA (78)	Simulation modelling study	Vehicle electrification	M	Premature mortality & death avoided	Spatial	(+)	(+-) Notes regional differences in CO2 emission reduction

Quilty et al., (2023), Australia (79)	Epidemiological Study - Case Time Series	Heat adaptation - physiological, sociocultural, and technological and infrastructural	A	Mortality	Indigenous population	(~)	(~) Despite marked socioeconomic inequality, the Aboriginal population was not observed to be more susceptible to heat mortality than the non-indigenous population
Richardson et al., (2012), USA (80)	Health Impact Assessment	Cap-and-Trade Regulations	M	Health generally	Spatial & SES	(+)	(-) Offset options may lead to negative determinants of health that disproportionately affect disadvantaged populations
Romitti et al., (2022), USA (81)	Multilevel mixed model	Air Conditioning	A	Heat exposure	Social Vulnerability	(+)	(-) AC distribution correlates with multiple social vulnerability indicators and summer daytime UHI effect intensity, highlighting exacerbated exposure to extreme heat for vulnerable groups.<br>(~) However, elderly residents are found to be more likely to have AC, but this is a weaker statistical relationship.
Sabel et al., (2016), Europe and	Modelling study	Urban greenhouse gas reduction policies: focused on	M	DALYs, physical health, disease & mortality	Spatial & SES	(+ - ~)	(+ -) Some interventions (e.g. reducing AC use) may have negative impacts on health of the general population but

China (82)		different fuels used in power generation, energy efficient buildings and transport modes					reduce health inequalities, because they are typically used by more affluent groups.<br><br>Interventions that reduce indoor air quality (e.g. insulation without ventilation) could widen health inequalities
Santamouris et al., (2020), Australia (83)	Modelling study	Heat mitigation technologies: reflective pavements & roofs, greenery in open spaces and buildings	M & A	Heat-related morbidity & mortality	Income	(+)	(+) positive impacts for low-income household
Sergi et al., (2020), USA (84)	Modelling study	Improving air quality by replacing power plants with new wind, solar, or natural gas	M	Premature mortality & reduced morbidity	Income	(+)	(+)Highest benefits in low-income households (lowest 60% relative to highest 20%)
Stone et al., (2023), USA (85)	Modelling Study	Heat management strategies including energy efficiency	M & A	Heat-related mortality	Spatial	(+)	(+) Largest benefits felt in the neighbourhoods with most to gain i.e. highest levels of heat-related mortality

		measures (buildings and vehicles) and urban cooling e.g. green infrastructure, cool roofs and pavements. 'Heat Health Maps' inform the targeting of interventions.					
Stowell et al., (2017), USA (86)	Modelling study	Emission control policies on O3 levels	M	Excess mortality	Spatial	(+)	(-) The West, Southeast, and Northeast regions showing the largest impact
Strid et al., (2023a), Sweden (87)	Cohort Study	Dietary shifts to lower climate impact diets	M	MI (Myocardial infarction/heart attack) or stroke diagnosis	Sex	(~)	(-) Some adverse health effects for men found when diet quality is not considered in the pursuit of more climate-sustainable diets. For women, no significant associations were detected. The mechanism underlying this association for men is noted to need further investigation.
Strid et al., (2023b),	Longitudinal	Diets with lower	M	All-cause	Sex	(+).	(~) no effect found

Sweden (88)	Cohort Study	GHGEs		mortality			
Stroud et al., (2022), USA (89)	Case Study Evaluation	Nature Based Solutions to mimimise flood impacts	M & A	Monetized values of co-benefits, including 'Health' in general, specifically 'Mental Stress'	SES, Social Vulnerability	(+)	(+) overall, though the paper makes some qualification<br><br>The value of NBS is felt more highly in the deprived neighbourhood, suggesting NBS can reduce inequalities and contribute to climate justice within an unequal city. Socially vulnerable neighbourhood has more to gain. However, some caveats remain, policies will be more effective on inequality if they protect against consequences such as gentrification (e.g. by providing affordable housing).
Tieges et al., (2020), Scotland (90)	Cohort	Canal regeneration	M & A	All-cause mortality	SES	(+)	(+) Better health outcomes for areas close and distant from the canal shows change in unequal distribution of mortality rates
van den Bogerd et	Conceptual	Green	M & A	Healthy Child	Age (paper looks at child	(+)	(+-) Positive impact on children's health, but the paper

al., (2023), The Netherlands (91)	Framework	Schoolyards		Development	wellbeing) Socio-economic inequality noted but not discussed in depth		notes that green infrastructures and schoolyards are unevenly distributed, being more common in privileged districts
Venter et al., (2020), Norway (92)	Modelling study	Green infrastructure: tree-cover, peri-urban forests, street trees & green space	M & A	Heat stress	Adults ages 65+	NA	(+) Reduction in morbidity in elderly people
Vernon & Jarvis (2011), Global (93)	Summary	Mitigation options in five economic sectors, including transport, agriculture, housing, household energy	M	Health gains	NA	(+)	(+) Health equity is noted as important
Willand et al., (2019), Australia (94)	Mixed methods: Quantitative & Qualitative	Residential energy efficiency	A	Health generally	Low income & elderly population	NA	(+) Health & wellbeing benefits (i.e. lower energy costs, keeping warm indoors, reduced involuntary air exchange, energy conservation, less moisture risk) experienced by

							target group
Williams et al., (2018), UK (95)	Modelling study	Carbon emission & air pollution mitigation action	M	Respiratory deaths, & all-cause mortality	SES	(+)	(~) despite substantial reductions in air pollution the most socioeconomically deprived are still exposed to higher concentrations
Wolf et al., (2014), Europe (96)	Report	Policies to improve air quality, transport and decarbonization of energy supply systems, dietary choices and sustainable diets, healthy and low carbon housing, protection of extreme weather events and vector borne diseases	M	Health co-benefits	Vulnerable populations	(+ -)	(+) Improved air quality & health co-benefits of reduced energy consumption & emissions has largest benefits for deprived and vulnerable populations.<br><br>Sustainable transport policies can be inclusive & accessible to groups normally excluded.<br><br>Sustainable diets which can be healthier for individuals allow for more equitable distribution/pricing of food globally.
Woodward et al., (2023), New Zealand	Report	Green spaces and trees in urban settings	M & A	General Health	Socioeconomic	(+ -)	(+) Paper recommends prioritising the hottest, least green spaces (using heat mapping and equitable planning/response processes),

(97)							as the greening of these areas will have the greatest impact on health inequalities. Disparities between wealthier suburbs and poorer suburbs regarding tree cover are well-documented, so these neighbourhoods have most to gain.
Yin et al., (2023), USA (98)	Case Study of Los Angeles	Urban Greening, increasing urban tree cover and surface albedo, greywater recycling, solar reflective cool pavements and roofs	M & A	Heat Exposure	Income Groups	(+)	(-) Current levels of albedo and greening are potentially exacerbating health inequalities because they are more prominent in affluent, high-income neighbourhoods. Low-income LA residents are more exposed to extreme heat.
Zhu et al., (2022), USA (99)	Modelling study	Decarbonization via truck electrification and building electrification	M	PM2.5 exposure	Socioeconomic, Level of community disadvantage, Ethnicity	(+)	(+) The building electrification achieves ~15% greater total health benefits than the truck electrification strategy, which uses renewable fuels to meet building demands. Conversely, the enhanced electrification of the truck sector is shown to benefit DACs [socially and economically disadvantaged

							communities] more effectively.<br><br>In both scenarios, the most disadvantaged groups experience a greater share of the health benefits, but this disproportionality is more pronounced in the truck electrification scenario.

Table 3b: Review papers

Author, Year & Country	Study Design	Climate Action	Mitigation and/or Adaptation	Health Outcome	Inequality Measure	Impact on Health	Impact on Health Inequality
Ambasta et al., (2018), Canada (100)	Review	Carbon pricing	M	Cardiovascular diseases, lung diseases, neurodegenerative diseases,& air pollution mortality rate	Socioeconomic & Indigenous communities	(+)	(-) if carbon pricing makes essential goods more expensive
Angotti et al., (2015),	Review	Urban agriculture	M & A	Health generally	Low-income communities	(+ -)	(+) revitalisation of low-income communities

USA (101)							
Barrett et al., (2022), USA (102)	Review	Adoption of sustainable diets	M	Morbidity, mortality & monetised health benefits	Gender	(+)	(-) Greater benefits for females compared with males
Bennett et al., (2014), New Zealand (103)	Review	Active transport, healthy eating, energy efficiency and moving away from fossil fuels	M & A	Heart disease, cancer, obesity, musculoskeletal disease, Type 2 diabetes, respiratory disease, motor vehicle injuries, & mental health	Low income and ethnic minorities (Maori and Pacific)	(+)	(+) for active transport & energy efficiency/improving indoor environments; not discussed for other actions
Berry et al., (2010), Australia (104)	Review	Caring for country projects	A-	Wellbeing	Focuses on Aboriginal Australians	NA	(+) Projects directly associated with improved health of Aboriginal Australians (but it is not known why this is or whether caring for country project outcomes would differ across climate zones and thus be generalisable)
Bikomeye et al., (2021b), Global (105)	Review and concep	Reducing Greenhouse Gas Emissions (limiting	M & A	Respiratory disease, mental & social wellbeing,	Age, Sex, Disability & SES	(+)	(+) Urban greenspaces offer additional opportunities for children to recess in greener

	tual framew ork	CO_2 increasing renewables and carbon sinks and reducing fossil fuels)		chronic diseases, heat-related illnesses, nutritional status, infectious diseases & injuries			and healthier schoolyards which improves a range of health outcomes and physical activity. Public transport enhances health equity by improving mobility for women, children, older adults, people with disabilities and the poor who have less access to private vehicles.
Bowen & Lynch (2017), Global (106)	Review	Green Infrastructure	M & A	Physical & mental health, wellbeing, cardiovascular & respiratory disease & mental stress	Socioeconomic, Elderly & people from culturally and linguistically diverse (CALD) backgrounds	(+)	(+) Reduced risk from heatwaves for vulnerable populations. Lower depression & anxiety for low SES populations.
Castillo et al., (2021), Global (107)	Review	Greenhouse Gas Mitigation Action to reduce air pollution, noise, increase green space and increase physical activity	M & A	Wide range of physical health outcomes (e.g., all-cause mortality, preterm/low birth weight, diabetes incidence, respiratory mortality, lung	SES, Age & Sex, Ethnicity	(+)	(+) Green space can have positive impacts for marginalised groups with proper foresight, regulation and community buy-in

				cancer, cardiovascular mortality, chronic obstructive pulmonary disease (COPD))			
Cleghorn et al., (2022), New Zealand (108)	Systematic Review & Modelling Study	Greenhouse Gas emission taxes on food	M	Quality Adjusted Life Years (QALYs) & net Health System Cost Savings	Ethnicity & Sex	(+)	(+) Health gains for Maori were bigger than for non-Maori in all four policies modelled
Cheng & Berry (2013), Global (109)	Review	Urban design and planning, & community outreach	A	Mental health, heat-related illness & mortality, cardio-respiratory disease, physical activity & allergies	Vulnerable groups (Elderly, people with chronic illness & the socially disadvantaged)	(+ -)	(+) social networks in vulnerable groups could facilitate adaptation to extreme temperatures
D'Amato et al., (2018), Global (110)	Review	Air conditioning	A	Respiratory health	Respiratory (vulnerable) patients	(-)	(-) AC spreads bacteria, viruses, allergens, fungal spores – disproportionately impacting vulnerable patients
Dannenberg et al.,	Review	Planned relocation of small communities at	A	Mental health & wellbeing, food	Indigenous & low-income	(+ -)	(-) Affected populations are predominantly low-income,

(2019), USA, Panama, Fiji, Papua New Guinea, Solomon Islands (111)		risk of sea level rise		security & nutrition, infectious disease risk, injury & access to healthcare			indigenous people. Effects include greater exposure to disease, reduced access to healthcare, potential loss of food sources & water security/sanitation.
Demuzere et al., (2014), Global (112)	Review	Green urban infrastructure	M & A	Health generally	Age, SES & Disability	(+)	(+) for children and those of a lower socioeconomic status
Deng et al., (2017), Global (113)	Review	Policies which aim to reduce air pollution (e.g., carbon tax, transitions to alternate fuel sources)	M	Health generally	Low-income households	(+)	(-) if revenue from taxes is not well-used lower income groups could be negatively affected
Dhar et al., (2009), New Zealand (114)	Review	Carbon pricing	M	Health generally	SES	(+)	(-) on poorer groups if taxes are regressive and cause the price of products and services to increase
Dwivedi et al., (2017), Global (115)	Review	Diversifying food systems using innovative plant breeding	M	Healthy diets & health generally	SES, age & sex	(+)	(-) there may be some differences in uptake of healthy diets by SES, age & sex
Egger (2008), Australia	Review	Personal Carbon Trading	M	Obesity	Age	(+)	(+) carbon costs of inactivity expected to increase activity

(116)							levels in children
Elmqvist et al., (2015), USA, Canada & China (117)	Review	Urban ecosystem services (greenspace)	M & A	Wellbeing	Age	(+)	(+) improves children's wellbeing as well as adults
Farbotko & Waitt (2011), Australia (118)	Review & case studies	Residential air conditioning	A	Wellbeing	Age, disability & income	(-)	(-) Lower-income groups will be more impacted by the increased electricity prices associated with the increased demand for air-conditioning. Older populations and those who are physically more vulnerable to heat stress will be negatively impacted by the increased GHG emissions/increased temperatures associated with more residential air conditioning, and the narrowing tolerance for a range of temperatures.
Fisk (2015), USA & Europe (119)	Review	Improved building energy efficiency & air conditioning	A	Respiratory health, chronic health effects, asthma &	Age & SES	(+ -)	(+) if these measures are targeted at vulnerable groups (e.g., elderly & low income)

				respiratory symptoms, hospitalisations, mortality, eye & nose symptoms, chronic obstructive pulmonary disease, allergies, colds and flu, wheezing, sleep disturbance, visits to GP, missed days of work & school due to sickness.			
Gulyas & Edmondson (2021), Global North (120)	Systematic Review	Urban agriculture	A	Access to nutritious foods & health risks associated with air pollution and ingestion of heavy metals	Socio-economically disadvantaged groups	(+ -)	(+) increase access to nutritious foods
Halsnæs et al., (2023), Denmark	Review	Various, including: Afforestation, Biomass production, Electric Vehicles,	M	General health, access to health care	Income, Disadvantaged groups (i.e. race, ethnicity,	(+)	(+-) Implementation/design of interventions is key otherwise there is a risk of replicating or exacerbating existing health

(121)		Public Transport, Urban Actions (green spaces and urban planning), Decarbonizing industry			socioeconomic status)		inequality
Holmner et al., (2012), Global (122)	Review	E-Health Interventions	M & A	Respiratory & cardiovascular diseases, access to healthcare, hospital admissions, & health economic costs	People with chronic diseases & rurality	(+)	(+) Provides health education and support to vulnerable regions with low densities of health workers
Hu et al., (2022), USA, Australia, China, Japan, Spain, New Zealand & UK (123)	Systematic Review	Improving residential ventilation and installing heating, High efficiency particulate air (HEPA) cleaners, & Long-term preventive medication to manage asthma	A	Childhood asthma symptoms, school absences, visits to doctor, visits to pharmacist & exacerbation of respiratory symptoms	Focused on children	NA	(+) Effective ventilation, heating, and air cleaning (alongside appropriate asthma medication) are found to improve asthmatic incidents among this vulnerable group, but several other adaptation measures are unknown/unclear at this stage.
Issac et al.,	Review	Disaster	A	Appropriate care',	Stroke	(+)	(+) Tailored advice and support

(2023), USA, Latin America & The Caribbean (124)		preparedness support (particularly hurricanes) for stroke survivors		'Life', 'Stroke prevention, control of comorbit conditions, or optimal functioning', 'Risk of COVID-19 and other infections'.	survivors		for at-risk population (stroke survivors) during a hurricane mitigates the likelihood of disproportionate or exacerbated health harms within this vulnerable group
Jay et al., (2021), Australia & USA (125)	Review	Air conditioning	A	Heat-strain, heat-related fatalities, thermal discomfort, physiological & perceptual adaptation, & heat stroke	Income & Age	(+ -)	(+) for children
Jennings et al., (2020), UK (126)	Review	Housing retrofit, active transport over motorised transport, decarbonising transport sector, sustainable diets & access to greenspace	M & A	Healthcare costs and NHS expenditure; physical and mental health, and wellbeing; risk of physical and mental illness; average life expectancy at	Age	(+ -)	(+) elderly and children most at risk for heat stress

				birth; YLL; incidence of obesity and type 2 diabetes; deficient in the micronutrients; treatment for anxiety or mood disorders; levels of stress; heat stress; admissions to the NHS			
Jones (2018), Wales, UK (127)	Review	Electric cars	M	Road traffic fatalities, pollution-related mortality, physical inactivity, obesity, stress, anxiety, hypertension & sleep loss	Age & Disability	(+ ~-)	(-) Negative health impacts for young, old and disabled people.
Kardan et al., (2023), Germany (128)	Scoping Review	Active Travel - Cycling	M	General Health	Age (over 60s)	(+)	(+) Older adults can often face barriers to cycling, interventions that remove these barriers (road safety measures, traffic calming, cycling promotion/education)

							can encourage more older adults to become physically active and improve their health - potentially reducing inequalities
Kim et al., (2023a), Japan (129)	Review	Japan's Climate Change Act and National Adaptation Plan, involving mitigation of anthropogenic heat and adaptations to prevent heat-related illness e.g. Air Conditioning	M & A	Heat Stress, Heat Exposure, Excess Deaths	Vulnerability, Age, SES	(+-)	(-) Living without air conditioning is associated with older adults and lower socioeconomic status.<br>(+) The paper highlights a pilot programme offering AC subscription to vulnerable households, though this is yet to be evaluated.
Kime et al., (2023), USA (130)	Review	Decarbonisation of the energy sector	M	Proximity to health hazard (e.g. fossil fuel infrastructure)<br><br>Pollutant Exposure (e.g. occupational pollutant concentration)<br><br>Health outcome	Ethnicity, income status<br><br>This paper includes 'health' as an outcome of 'equity status'	(+)	(-) Renewable energy incentives typically benefit high-income households more<br>(-) EVs require high up-front costs, typically benefiting high-income households<br>(+) Fossil fuel phaseouts/Power plant decommissions: projected health benefits greater in disadvantaged neighbourhoods

				(e.g. avoided premature mortality, incidence rates of diseases)<br><br>Health monetization (e.g. monetized health benefits or costs $)			(+) Residential electrification: positive health impact for asthma in black and low-income children
Lake et al., (2012), UK (131)	Review	Changes in food consumption in response to changes in availability of foods due to climate change	A	Health generally, nutritional deficits, obesity, & food-transmitted pathogens	SES	(-)	(-) low socioeconomic status groups more at risk of adaptation behaviours with risk to health
Lake et al., (2018), UK (132)	Review	Increased surveillance & governance of foodborne pathogens	A	Foodborne illnesses and associated deaths	Age, Sex & those with limited immune response	(+)	(+) Not covered in detail but one named outcome of the public health approach is user-centric communications strategies to engage diverse stakeholder groups and the need to target the elderly, pregnant women and people with limited immune responses.

Leder et al., (2002), Australia (133)	Review	Use of alternative (untreated) water sources	M & A	Health generally	Geographical, rurality & indigenous communities	(-)	(-) Additional challenges of managing scarce and safe water resources in particularly remote communities
Lenzer et al., (2020), Germany (134)	Scopin g Review	Air conditioning	A	Blood pressure, mortality, overheating, survival of heat-related illness, healing of wounds, cardiac function & effects on existing conditions	Age	(+ -)	(+) Older patients more at risk from heat-related illness
Linares et al., (2020), Spain (135)	Review	Preparedness & adaptation within health systems including Health Action Plans	A	Health risks from heat or drought, climate sensitive diseases & mortality	Geographical, SES, Age & Sex	(+)	(+) Noted in the paper that vulnerable and at-risk groups should be prioritised
Lowe (2014), Australia (136)	Review	Policies aimed at both reducing obesity & climate change (e.g., active travel, improving diets & food systems)	M	Obesity and associated conditions (e.g., type 2 diabetes) & wellbeing	SES, Ethnicity & Indigenous populations	(+)	(+) Macroeconomic reforms to tackle structural causes of obesity and climate change lead to long-term health benefits and reduced inequalities

Lowe et al., (2011), Europe (137)	Scoping Review	Heatwave Early Warning Systems & additional mitigation action (e.g., green infrastructure, energy efficiency & renewable energy systems)	M & A	Health generally including morbidity & mortality	Age, SES & Disability	(+)	(-) Noted that some population may have higher accessibility barriers
Luyten et al., (2023), Global (138)	Scoping Review	Renewable energy, Fuel efficient vehicles, Electric vehicles, Fuel switching, Carbon Capture, Green infrastructure, Albedo increase, Early warning systems	M & A	Mortality and Morbidity measures, including DALYs, number of hospitalizations, number of intensive care admissions.	Age, Socio-economic variables	(+-)	(+-) Paper notes overall 'lack of health impact stratification', suggesting that identifying vulnerable groups could improve health equity.
Magkos et al., (2019), USA (139)	Review	Sustainable diets	M	Obesity	Income & Education	(+)	(-) Noted that not all groups will have the resources to adopt a plant-based diet that is also healthy
Mailloux et al., (2021), USA (140)	Review	Reducing the sources of emissions (e.g., sustainable diets, renewables), restoring & protecting carbon	M	Health generally, respiratory & cardiovascular disease, diabetes, malnutrition,	SES, Ethnicity, Spatial & Sex	(+)	(+-) Note that the impact on health inequality is influenced by how the interventions are carried out and for whom

		sinks, & health and education measures to target climate change		obesity, Ebola & malaria			
Markkanen & Anger-Kravi (2019), UK (141)	Review	Policies to reduce energy consumption, policies to increase the deployment of renewables, & policies to develop and preserve natural carbon sinks	M	General health & wellbeing	Economic, Sex & Ethnicity	(+ -)	(+ -) depending on contextual factors, policy design and policy implementation
McMurtry & Krogh (2014), Canada (142)	Review	Wind turbines	M	Symptoms including neurological, cognitive, cardiovascular, psychological, regulatory & systemic disorders	Spatial & SES	(-)	(-) Higher risk of adverse effects (health effects but also including displacement, rural poverty, landlessness) to rural populations
Milner et al., (2012), UK (143)	Review	Low carbon cities (e.g., energy efficiency in housing, interventions to encourage active	M	Chronic disease, mental wellbeing & physical injury	SES	(+)	(+) Health benefits felt in low-income settings e.g. through reduction in energy poverty

		travel and public transport use, & switching to low carbon sources of power)					
Nieuwenhuijsen & Khreis (2016), Spain (144)	Review	Car free cities	M	Morbidity & mortality	SES	(+ -)	(+) Improving public transport and active travel infrastructure can strengthen social capital by providing a safety net of transport options for (economically) disadvantaged groups<br><br>(-) Motorized traffic may detour around car free zones, displacing pollution effects and possibly exacerbating socioeconomic health divides
Nori-Sarma & Wellenius (2023), USA (145)	Narrative Review	Urban greening, Energy efficiency in buildings, Active Travel, Heat Action Plans (HAPs)	M & A	General Health	(Socio-economic) Vulnerability	(+)	(+) Health impacts could have largest impacts on disadvantaged populations because they have the most to gain e.g. opportunities for health benefits from urban greening in low-income urban communities, or specific targeting of HAPs

O'Neill et al., (2009), USA (146)	Review	Preventative strategies for heat: Heat Health Warning Systems, air conditioning, planting trees	M & A	Heat-related morbidity & mortality	Age, SES & those with chronic health conditions	(+ -)	(+) if targeted at vulnerable groups
Oliveira et al., (2015), Japan (147)	Review	Reducing carbon emissions by shifting to cleaner energy & increasing energy efficiency; transport, urban planning, waste & energy sectors	M & A	Morbidity, mortality, DALYs, and other health conditions	SES	(+)	(+) Targeting low-income households can have the greatest health improvements
Perera et al., (2019), USA (148)	Systematic Review	Reducing fossil fuel combustion	M	Pre-term birth, low birthweight, autism, attention deficit hyperactivity disorder, IQ reduction & the development of childhood asthma	Age: focuses on children only	NA	(+) Adverse health outcomes in children associated with pollutants are mitigated.<br>Not specifically considered, but the authors note that the least advantaged children are likely to suffer disproportionately the ill-effects of polluting emissions.
Picetti et al., (2023), Global (149)	Systematic Review	Greenhouse Gas Emission reductions e.g. Carbon	M	Medical visits, mortality, school absences,	Age (child and adolescent health)	(+)	(+) Child and adolescent health outcomes improve

		taxes/policies, Energy efficiency measures, Exhaust-free transport, Traffic reduction, Active travel		neurological, perinatal, and respiratory outcomes			
Rodgers et al., (2023), New Zealand (150)	Literature Review	Urban greening	M & A	Wellbeing	Indigenous population	(+)	(+) Benefits of this greening may be particularly felt by Maori populations, who live primarily in urban areas
Quam et al., (2017), Sweden (151)	Review	Lifestyle-related climate mitigation strategies (active travel & sustainable diets)	M	Morbidity, mortality, DALYs, weight gain, all-cause mortality, caloric & nutrient consumption	NA	(+)	(-~) Affordability of diets is identified as a barrier but not explored
Sharifi et al., (2021), Global (152)	Systematic Review	Measures for infrastructure, nature-based solutions, housing and building design, urban planning and design, early warning systems, policy/management	M & A	Health & wellbeing generally	NA	(+)	(+-) Noted that policy needs to consider the needs and priorities of different social groups

		and governance, knowledge/perceptions/behaviour					
Sharma et al., (2023), Global (153)	Review and synthesis	Electric Vehicles	M	Mortality, Respiratory conditions	Spatial	(+-)	(+ - ~) Mixed results for health inequalities.<br>Note that: 'very few studies quantified the disparities in air pollution and health' (less than a fifth of studies included in this review)
Shaw et al., (2014), Global (154)	Systematic Review	Mitigation policies that decrease transport sector (road, rail, aviation and maritime) CO_2 emissions	M	Health determinants & Health generally	SES, Ethnicity & Sex	(+)	(~) Noted that there was little focus on any inequalities in the included studies
Shonkoff et al., (2011), USA (155)	Review	Mitigation policies (e.g., tax on emissions, auctioning of emission credits, carbon-caps)	M	Premature mortality, hospital admissions for respiratory causes & cardiovascular disease, asthma and respiratory symptoms, acute bronchitis, work loss days & minor restricted activity	SES	(+)	(+ -) Benefits of reduced pollution felt by low-income groups and people of colour in segregated neighbourhoods (+), but this could be under-achieved by over-allocation of emission credits (-)<br>(-) Burden of costs disproportionately felt by low-income households

				days			
Sin et al., (2023), Global (156)	Review	Reducing emissions e.g. decarbonization of energy, public transport, active travel, fuel efficiency, soil restoration, carbon sequestration, Early Warning Systems, respiratory masks, Air cleaners/filters	M & A	COPD, Lung Disease	SES, Age, Spatial	(+)	(+-) Positive effect identified on age-related health inequality for long-term air quality improvement in LA: children's lung development (+)<br><br>Negative effect identified on income-related health inequality for traffic-related intervention in Rome: most air quality benefits experienced in high-income areas (-)
Smith et al., (2013), Global (157)	Review	Reducing fossil fuel use through energy efficiency and large-scale renewable technologies e.g. hydropower	M	Health risks	Vulnerable populations	(+)	(-) Construction of large dams has caused involuntary displacement of people that reside in areas to be flooded, often these are vulnerable populations, subject to social and health burdens as well as forced displacement.
Wang et al., (2016), Europe (158)	Systematic Review	Air pollution control strategies	M	Health generally, morbidity & mortality	SES	(+)	(~) The effectiveness of strategies to improve health equity was inconclusive

1. Achebak H, Rey G, Lloyd SJ, Quijal-Zamorano M, Fernando Méndez-Turrubiates R, Ballester J. Drivers of the time-varying heat-cold-mortality association in Spain: A longitudinal observational study. Environment International. 2023;182:108284.
2. Africa J, Heerwagen J, Loftness V, Ryan Balagtas C. Biophilic Design and Climate Change: Performance Parameters for Health. Frontiers in Built Environment. 2019;Volume 5 - 2019.
3. Anderson CM, Kissel KA, Field CB, Mach KJ. Climate Change Mitigation, Air Pollution, and Environmental Justice in California. Environmental Science & Technology. 2018;52(18):10829-38.
4. Anderson V, Gough WA, Agic B. Nature-Based Equity: An Assessment of the Public Health Impacts of Green Infrastructure in Ontario Canada. International Journal of Environmental Research and Public Health. 2021;18(11):5763.
5. Angradi TR, Launspach JJ, Wick MJ. Human well-being and natural capital indicators for Great Lakes waterfront revitalization. Journal of Great Lakes Research. 2022;48(4):1104-20.
6. Appolloni L, Dettori M, Petronio MG, Raffo M, Settimo G, Rebecchi A, et al. A proposal of hygienic and sanitary standards for the new Building Code in Italy. Ann Ig. 2020;32(5 Supple 1):85-109.
7. Arai R, Kiguchi M, Murakami M. A Quantitative Estimation of the Effects of Measures to Counter Climate Change on Well-Being: Focus on Non-Use of Air Conditioners as a Mitigation Measure in Japan. Sustainability. 2020;12(20):8694.
8. Asikainen A, Pärjälä E, Jantunen M, Tuomisto JT, Sabel, E. C. Effects of Local Greenhouse Gas Abatement Strategies on Air Pollutant Emissions and on Health in Kuopio, Finland. Climate. 2017;5(2):43.
9. Astell-Burt T, Navakatikyan MA, Feng X. Urban green space, tree canopy and 11-year risk of dementia in a cohort of 109,688 Australians. Environment International. 2020;145:106102.
10. Bailey J, Gerasopoulos E, Rojas-Rueda D, Benmarhnia T. Potential health and equity co-benefits related to the mitigation policies reducing air pollution from residential wood burning in Athens, Greece. J Environ Sci Health A Tox Hazard Subst Environ Eng. 2019;54(11):1144-51.
11. Baldwin C, Tony M, and Byrne J. Planning for Older People in a Rapidly Warming and Ageing World: The Role of Urban Greening. Urban Policy and Research. 2020;38(3):199-212.
12. Bell R, Khan M, Romeo-Velilla M, Stegeman I, Godfrey A, Taylor T, et al. Ten Lessons for Good Practice for the INHERIT Triple Win: Health, Equity, and Environmental Sustainability. International Journal of Environmental Research and Public Health. 2019;16(22):4546.
13. Bikomeye JC, Namin S, Anyanwu C, Rublee CS, Ferschinger J, Leinbach K, et al. Resilience and Equity in a Time of Crises: Investing in Public Urban Greenspace Is Now More Essential Than Ever in the US and Beyond. International Journal of Environmental Research and Public Health. 2021;18(16):8420.

14. Braubach M, Tobollik M, Mudu P, Hiscock R, Chapizanis D, Sarigiannis DA, et al. Development of a quantitative methodology to assess the impacts of urban transport interventions and related noise on well-being. Int J Environ Res Public Health. 2015;12(6):5792-814.
15. Buchin O, Hoelscher M-T, Meier F, Nehls T, Ziegler F. Evaluation of the health-risk reduction potential of countermeasures to urban heat islands. Energy and Buildings. 2016;114:27-37.
16. Burke M, Driscoll A, Heft-Neal S, Xue J, Burney J, Wara M. The changing risk and burden of wildfire in the United States. Proceedings of the National Academy of Sciences. 2021;118(2):e2011048118.
17. Burlotos A, Dresser C, Shandas V. Portland's Response to the Western North American Heatwave: A Brief Report. Disaster Med Public Health Prep. 2023;17:e522.
18. Carlton EJ, Barton K, Shrestha PM, Humphrey J, Newman LS, Adgate JL, et al. Relationships between home ventilation rates and respiratory health in the Colorado Home Energy Efficiency and Respiratory Health (CHEER) study. Environmental Research. 2019;169:297-307.
19. Chakraborty T, Biswas T, Campbell LS, Franklin B, Parker SS, Tukman M. Feasibility of afforestation as an equitable nature-based solution in urban areas. Sustainable Cities and Society. 2022;81:103826.
20. Chen D, Wang X, Thatcher M, Barnett G, Kachenko A, Prince R. Urban vegetation for reducing heat related mortality. Environmental pollution (Barking, Essex : 1987). 2014;192.
21. Chiabai A, Quiroga S, Martinez-Juarez P, Higgins S, Taylor T. The nexus between climate change, ecosystem services and human health: Towards a conceptual framework. Science of The Total Environment. 2018;635:1191-204.
22. Cifuentes L, Borja-Aburto VH, Gouveia N, Thurston G, Davis DL. Assessing the health benefits of urban air pollution reductions associated with climate change mitigation (2000-2020): Santiago, São Paulo, México City, and New York City. Environ Health Perspect. 2001;109 Suppl 3(Suppl 3):419-25.
23. de'Donato F, Scortichini M, De Sario M, de Martino A, Michelozzi P. Temporal variation in the effect of heat and the role of the Italian heat prevention plan. Public Health. 2018;161:154-62.
24. Dietz WH, Pryor S. How Can We Act to Mitigate the Global Syndemic of Obesity, Undernutrition, and Climate Change? Current Obesity Reports. 2022;11(3):61-9.
25. Dufty N. Using heat refuges in heatwave emergencies. Australian Journal of Emergency Management. 2022;37:38.
26. Farchi S, De Sario M, Lapucci E, Davoli M, Michelozzi P. Meat consumption reduction in Italian regions: Health co-benefits and decreases in GHG emissions. PLoS One. 2017;12(8):e0182960.
27. Ford JD, Sherman M, Berrang-Ford L, Llanos A, Carcamo C, Harper S, et al. Preparing for the health impacts of climate change in Indigenous communities: The role of community-based adaptation. Global Environmental Change. 2018;49:129-39.
28. Fraser AM, Chester MV. Transit system design and vulnerability of riders to heat. Journal of Transport & Health. 2017;4:216-25.

29. Fraser A, Chester M. Transit Planning and Climate Change: Reducing Rider's Vulnerability to Heat2017. 456-64 p.
30. Fratini F. The Eco-Pedagogical Microforest a shared oasis of proximity. A cutting-edge project at the intersection of ecology, urbanism and pedagogy. TeMA - Journal of Land Use, Mobility and Environment. 2023;0(2):33-54.
31. Ferrari M, Benvenuti L, Rossi L, De Santis A, Sette S, Martone D, et al. Could Dietary Goals and Climate Change Mitigation Be Achieved Through Optimized Diet? The Experience of Modeling the National Food Consumption Data in Italy. Frontiers in Nutrition. 2020;Volume 7 - 2020.
32. FitzRoy F, Franz-Vasdeki J, Papyrakis E. Climate Change Policy and Subjective Well-Being. Environmental Policy and Governance. 2012;22(3):205-16.
33. Friel S, Hancock T, Kjellstrom T, McGranahan G, Monge P, Roy J. Urban health inequities and the added pressure of climate change: an action-oriented research agenda. J Urban Health. 2011;88(5):886-95.
34. Fujimoto M, Hayashi K, Nishiura H. Possible adaptation measures for climate change in preventing heatstroke among older adults in Japan. Frontiers in Public Health. 2023;Volume 11 - 2023.
35. Gabbe CJ, Chang JS, Kamson M, Seo E. Reducing heat risk for people experiencing unsheltered homelessness. International Journal of Disaster Risk Reduction. 2023;96:103904.
36. Gallagher CL, Holloway T. U.S. decarbonization impacts on air quality and environmental justice. Environmental Research Letters. 2022;17(11):114018.
37. Garcia E, Johnston J, McConnell R, Palinkas L, Eckel SP. California's early transition to electric vehicles: Observed health and air quality co-benefits. Science of The Total Environment. 2023;867:161761.
38. Giles-Corti B, Foster S, Shilton T, Falconer R. The co-benefits for health of investing in active transportation. N S W Public Health Bull. 2010;21(5-6):122-7.
39. Gölitzer F, Barbir J, Eustachio JHPP. Saving energy at university campus via intervention to reduce elevator usage – a case study from Germany. Frontiers in Sustainability. 2023;Volume 4 - 2023.
40. Gong J, Part C, Hajat S. Current and future burdens of heat-related dementia hospital admissions in England. Environ Int. 2022;159:107027.
41. Green M HA, Main E, Early - Alberts J, Dubuisson N, Douglas JP. Climate Smart Communities Scenarios Health Impact Assessment. Portland, OR: Oregon Health Authority; 2013.
42. Grummon AH, Lee CJY, Robinson TN, Rimm EB, Rose D. Simple dietary substitutions can reduce carbon footprints and improve dietary quality across diverse segments of the US population. Nature Food. 2023;4(11):966-77.
43. de Guzman EB, Escobedo FJ, O'Leary R. A socio-ecological approach to align tree stewardship programs with public health benefits in marginalized neighborhoods in Los Angeles, USA. Frontiers in Sustainable Cities. 2022;Volume 4 - 2022.
44. Hanus NL, Wong-Parodi G, Vaishnav PT, Darghouth NR, Azevedo IL. Solar PV as a mitigation strategy for the US education sector. Environmental Research Letters. 2019;14(4):044004.

45. Hebbern C, Gosselin P, Chen K, Chen H, Cakmak S, MacDonald M, et al. Future temperature-related excess mortality under climate change and population aging scenarios in Canada. Can J Public Health. 2023;114(5):726-36.
46. Heudorf U, Schade M. Heat waves and mortality in Frankfurt am Main, Germany, 2003–2013. Zeitschrift für Gerontologie und Geriatrie. 2014;47(6):475-82.
47. Hochard J, Li Y, Abashidze N. Associations of hurricane exposure and forecasting with impaired birth outcomes. Nature Communications. 2022;13(1):6746.
48. Hoffmann C, Liebers U, Humbsch P, Drozdek M, Bölke G, Hoffmann P, et al. An adaptation strategy to urban heat: hospital rooms with radiant cooling accelerate patient recovery. ERJ Open Res. 2021;7(3).
49. Izquierdo R, García Dos Santos S, Borge R, Paz Ddl, Sarigiannis D, Gotti A, et al. Health impact assessment by the implementation of Madrid City air-quality plan in 2020. Environmental Research. 2020;183:109021.
50. Jee SH, Friedman E, Etzel RA, Nguyen VT, Sack TL, Kemper KJ. Climate Change Imperils Pediatric Health: Child Advocacy Through Fossil Fuel Divestment. Yale J Biol Med. 2023;96(2):233-9.
51. Johnson S, Haney J, Cairone L, Huskey C, Kheirbek I. Assessing Air Quality and Public Health Benefits of New York City's Climate Action Plans. Environmental Science & Technology. 2020;54(16):9804-13.
52. Johnson DE, Fisher K, Parsons M. Diversifying Indigenous Vulnerability and Adaptation: An Intersectional Reading of Māori Women's Experiences of Health, Wellbeing, and Climate Change. Sustainability. 2022;14(9):5452.
53. Johnson D, Parsons M, Fisher K. Adaptation at whose expense? Explicating the maladaptive potential of water storage and climate-resilient growth for Māori women in northern Aotearoa. Global Environmental Change. 2023;82:102733.
54. Kabisch N, Frantzeskaki N, Pauleit S, Naumann S, Davis M, Artmann M, et al. Nature-based solutions to climate change mitigation and adaptation in urban areas

perspectives on indicators, knowledge gaps, barriers, and opportunities for action. Ecology and Society. 2016;21(2).
55. Karakas F, Grassie D, Schwartz Y, Dong J, Chalabi Z, Mumovic D, et al. School building energy efficiency and NO2 related risk of childhood asthma in England and Wales: Modelling study. Science of The Total Environment. 2023;901:166109.
56. Kim YJ, Park C, Lee DK, Park TY. Connecting public health with urban planning: allocating walkable cooling shelters considering older people. Landscape and Ecological Engineering. 2023;19(2):257-69.
57. Kingsborough A, Jenkins K, Hall JW. Development and appraisal of long-term adaptation pathways for managing heat-risk in London. Climate Risk Management. 2017;16:73-92.
58. Klopfer F, Pfeiffer A. Determining spatial disparities and similarities regarding heat exposure, green provision, and social structure of urban areas - A study on the city district level in the Ruhr area, Germany. Heliyon. 2023;9(6).
59. Konijnendijk CC. Evidence-based guidelines for greener, healthier, more resilient neighbourhoods: Introducing the 3–30–300 rule. Journal of Forestry Research. 2023;34(3):821-30.

60. Hayes CKaS. The value of energy efficiency as a public health and climate mitigation strategy. ECEEE: European Council for an Energy Efficient Economy; 2019.
61. Kuchcik M, Dudek W, Błażejczyk K, Milewski P, Błażejczyk A. Two faces to the greenery on housing estates–mitigating climate but aggravating allergy. A Warsaw case study. Urban Forestry & Urban Greening. 2016;16:170-81.
62. Lane K, Smalls-Mantey L, Hernández D, Watson S, Jessel S, Jack D, et al. Extreme Heat and COVID-19 in New York City: An Evaluation of a Large Air Conditioner Distribution Program to Address Compounded Public Health Risks in Summer 2020. Journal of Urban Health. 2023;100(2):290-302.
63. Long Y, Wu Y, Xie Y, Huang L, Wang W, Liu X, et al. PM2.5 and ozone pollution-related health challenges in Japan with regards to climate change. Global Environmental Change. 2023;79:102640.
64. Lowe R, García-Díez M, Ballester J, Creswick J, Robine JM, Herrmann FR, et al. Evaluation of an Early-Warning System for Heat Wave-Related Mortality in Europe: Implications for Sub-seasonal to Seasonal Forecasting and Climate Services. Int J Environ Res Public Health. 2016;13(2):206.
65. Lucas K, Pangbourne K. Assessing the equity of carbon mitigation policies for transport in Scotland. Case Studies on Transport Policy. 2014;2(2):70-80.
66. Luo Q, Copeland B, Garcia-Menendez F, Johnson JX. Diverse Pathways for Power Sector Decarbonization in Texas Yield Health Cobenefits but Fail to Alleviate Air Pollution Exposure Inequities. Environmental Science & Technology. 2022;56(18):13274-83.
67. Mailloux NA, Abel DW, Holloway T, Patz JA. Nationwide and Regional PM2.5-Related Air Quality Health Benefits From the Removal of Energy-Related Emissions in the United States. GeoHealth. 2022;6(5):e2022GH000603.
68. Marí-Dell’Olmo M, Oliveras L, Barón-Miras LE, Borrell C, Montalvo T, Ariza C, et al. Climate Change and Health in Urban Areas with a Mediterranean Climate: A Conceptual Framework with a Social and Climate Justice Approach. International Journal of Environmental Research and Public Health. 2022;19(19):12764.
69. Marvuglia A, Koppelaar R, Rugani B. The effect of green roofs on the reduction of mortality due to heatwaves: Results from the application of a spatial microsimulation model to four European cities. Ecological Modelling. 2020;438:109351.
70. McMichael AJ, Kovats RS. Climate Change and Climate Variability: Adaptations to Reduce Adverse Health Impacts. Environmental Monitoring and Assessment. 2000;61(1):49-64.
71. Meyerricks S, White RM. Communities on a Threshold: Climate Action and Wellbeing Potentialities in Scotland. Sustainability. 2021;13(13):7357.
72. O’Neill BC, M. Done J, Gettelman A, Lawrence P, Lehner F, Lamarque J-F, et al. The Benefits of Reduced Anthropogenic Climate changE (BRACE): a synthesis. Climatic Change. 2018;146(3):287-301.
73. Oka K, Honda Y, Phung VLH, Hijioka Y. Prediction of climate change impacts on heatstroke cases in Japan's 47 prefectures with the effect of long-term heat adaptation. Environ Res. 2023;232:116390.

74. Ortiz LE, Stiles R, Whitaker S, Maibach E, Kinter J, Henneman L, et al. Public health benefits of zero-emission electric power generation in Virginia. Heliyon. 2023;9(9):e20198.
75. Patterson E, Eustachio Colombo P, Milner J, Green R, Elinder LS. Potential health impact of increasing adoption of sustainable dietary practices in Sweden. BMC Public Health. 2021;21(1):1332.
76. Perera F, Cooley D, Berberian A, Mills D, Kinney P. Co-Benefits to Children's Health of the U.S. Regional Greenhouse Gas Initiative. Environmental Health Perspectives. 2020;128(7):077006.
77. Perez L, Trüeb S, Cowie H, Keuken MP, Mudu P, Ragettli MS, et al. Transport-related measures to mitigate climate change in Basel, Switzerland: A health-effectiveness comparison study. Environ Int. 2015;85:111-9.
78. Peters DR, Schnell JL, Kinney PL, Naik V, Horton DE. Public Health and Climate Benefits and Trade-Offs of U.S. Vehicle Electrification. GeoHealth. 2020;4(10):e2020GH000275.
79. Quilty S, Jupurrurla NF, Lal A, Matthews V, Gasparrini A, Hope P, et al. The relative value of sociocultural and infrastructural adaptations to heat in a very hot climate in northern Australia: a case time series of heat-associated mortality. The Lancet Planetary Health. 2023;7(8):e684-e93.
80. Richardson MJ, English P, Rudolph L. A health impact assessment of California's proposed cap-and-trade regulations. Am J Public Health. 2012;102(9):e52-8.
81. Romitti Y, Sue Wing I, Spangler KR, Wellenius GA. Inequality in the availability of residential air conditioning across 115 US metropolitan areas. PNAS Nexus. 2022;1(4).
82. Sabel CE, Hiscock R, Asikainen A, Bi J, Depledge M, van den Elshout S, et al. Public health impacts of city policies to reduce climate change: findings from the URGENCHE EU-China project. Environ Health. 2016;15 Suppl 1(Suppl 1):25.
83. Santamouris M, Paolini R, Haddad S, Synnefa A, Garshasbi S, Hatvani-Kovacs G, et al. Heat mitigation technologies can improve sustainability in cities. An holistic experimental and numerical impact assessment of urban overheating and related heat mitigation strategies on energy consumption, indoor comfort, vulnerability and heat-related mortality and morbidity in cities. Energy and Buildings. 2020;217:110002.
84. Sergi BJ, Adams PJ, Muller NZ, Robinson AL, Davis SJ, Marshall JD, et al. Optimizing Emissions Reductions from the U.S. Power Sector for Climate and Health Benefits. Environmental Science & Technology. 2020;54(12):7513-23.
85. Stone B, Lanza K, Mallen E, Vargo J, Russell A. Urban Heat Management in Louisville, Kentucky: A Framework for Climate Adaptation Planning. Journal of Planning Education and Research. 2023;43(2):346-58.
86. Stowell JD, Kim Y-m, Gao Y, Fu JS, Chang HH, Liu Y. The impact of climate change and emissions control on future ozone levels: Implications for human health. Environment International. 2017;108:41-50.
87. Strid A, Johansson I, Lindahl B, Hallström E, Winkvist A. Toward a More Climate-Sustainable Diet: Possible Deleterious Impacts on Health When Diet Quality Is Ignored. The Journal of Nutrition. 2023;153(1):242-52.

88. Strid A, Hallström E, Lindroos AK, Lindahl B, Johansson I, Winkvist A. Adherence to the Swedish dietary guidelines and the impact on mortality and climate in a population-based cohort study. Public Health Nutrition. 2023;26(11):2333-42.
89. Stroud HM, Kirshen PH, Timmons D. Monetary evaluation of co-benefits of nature-based flood risk reduction infrastructure to promote climate justice. Mitigation and Adaptation Strategies for Global Change. 2022;28(1):5.
90. Tieges Z, McGregor D, Georgiou M, Smith N, Saunders J, Millar R, et al. The Impact of Regeneration and Climate Adaptations of Urban Green–Blue Assets on All-Cause Mortality: A 17-Year Longitudinal Study. International Journal of Environmental Research and Public Health. 2020;17(12):4577.
91. van den Bogerd N, Hovinga D, Hiemstra JA, Maas J. The Potential of Green Schoolyards for Healthy Child Development: A Conceptual Framework. Forests. 2023;14(4):660.
92. Venter ZS, Krog NH, Barton DN. Linking green infrastructure to urban heat and human health risk mitigation in Oslo, Norway. Science of The Total Environment. 2020;709:136193.
93. Vernon W, Jarvis A. A Synopsis of Health in the Green Economy: How Carbon Reduction May Impact Health in Health Sector Services. Proceedings of the 2011 IEEE Global Humanitarian Technology Conference: IEEE Computer Society; 2011. p. 127–30.
94. Willand N, Maller C, Ridley I. Addressing health and equity in residential low carbon transitions – Insights from a pragmatic retrofit evaluation in Australia. Energy Research & Social Science. 2019;53:68-84.
95. Williams ML, Lott MC, Kitwiroon N, Dajnak D, Walton H, Holland M, et al. The Lancet Countdown on health benefits from the UK Climate Change Act: a modelling study for Great Britain. The Lancet Planetary Health. 2018;2(5):e202-e13.
96. Organization WH. Protecting health in Europe from climate change: 2017 update. World Health Organisation; 2017.
97. Woodward A, Hinwood A, Bennett D, Grear B, Vardoulakis S, Lalchandani N, et al. Trees, Climate Change, and Health: An Urban Planning, Greening and Implementation Perspective. Int J Environ Res Public Health. 2023;20(18).
98. Yin Y, He L, Wennberg PO, Frankenberg C. Unequal exposure to heatwaves in Los Angeles: Impact of uneven green spaces. Science Advances. 2023;9(17):eade8501.
99. Zhu S, Mac Kinnon M, Carlos-Carlos A, Davis SJ, Samuelsen S. Decarbonization will lead to more equitable air quality in California. Nature Communications. 2022;13(1):5738.
100. Ambasta A, Buonocore JJ. Carbon pricing: a win-win environmental and public health policy. Canadian Journal of Public Health. 2018;109(5):779-81.
101. Angotti T. Urban agriculture: long-term strategy or impossible dream?: Lessons from Prospect Farm in Brooklyn, New York. Public Health. 2015;129(4):336-41.
102. Barrett B. Health and sustainability co-benefits of eating behaviors: Towards a science of dietary eco-wellness. Preventive Medicine Reports. 2022;28:101878.

103. Bennett H, Jones R, Keating G, Woodward A, Hales S, Metcalfe S. Health and equity impacts of climate change in Aotearoa-New Zealand, and health gains from climate action. N Z Med J. 2014;127(1406):16-31.
104. Berry HL, Butler JR, Burgess CP, King UG, Tsey K, Cadet-James YL, et al. Mind, body, spirit: co-benefits for mental health from climate change adaptation and caring for country in remote Aboriginal Australian communities. N S W Public Health Bull. 2010;21(5-6):139-45.
105. Bikomeye JC, Rublee CS, Beyer KMM. Positive Externalities of Climate Change Mitigation and Adaptation for Human Health: A Review and Conceptual Framework for Public Health Research. International Journal of Environmental Research and Public Health. 2021;18(5):2481.
106. Bowen KJ, Lynch Y. The public health benefits of green infrastructure: the potential of economic framing for enhanced decision-making. Current Opinion in Environmental Sustainability. 2017;25:90-5.
107. Castillo MD, Anenberg SC, Chafe ZA, Huxley R, Johnson LS, Kheirbek I, et al. Quantifying the Health Benefits of Urban Climate Mitigation Actions: Current State of the Epidemiological Evidence and Application in Health Impact Assessments. Frontiers in Sustainable Cities. 2021;Volume 3 - 2021.
108. Cleghorn C, Mulder I, Macmillan A, Mizdrak A, Drew J, Nghiem N, et al. Can a Greenhouse Gas Emissions Tax on Food also Be Healthy and Equitable? A Systemised Review and Modelling Study from Aotearoa New Zealand. International Journal of Environmental Research and Public Health. 2022;19(8):4421.
109. Cheng JJ, Berry P. Health co-benefits and risks of public health adaptation strategies to climate change: a review of current literature. Int J Public Health. 2013;58(2):305-11.
110. D’Amato M, Molino A, Calabrese G, Cecchi L, Annesi-Maesano I, D’Amato G. The impact of cold on the respiratory tract and its consequences to respiratory health. Clinical and Translational Allergy. 2018;8(1):20.
111. Dannenberg AL, Frumkin H, Hess JJ, Ebi KL. Managed retreat as a strategy for climate change adaptation in small communities: public health implications. Climatic Change. 2019;153(1):1-14.
112. Demuzere M, Orru K, Heidrich O, Olazabal E, Geneletti D, Orru H, et al. Mitigating and adapting to climate change: Multi-functional and multi-scale assessment of green urban infrastructure. Journal of Environmental Management. 2014;146:107-15.
113. Deng H-M, Liang Q-M, Liu L-J, Anadon LD. Co-benefits of greenhouse gas mitigation: a review and classification by type, mitigation sector, and geography. Environmental Research Letters. 2017;12(12):123001.
114. Dhar D, Macmillan A, Lindsay G, Woodward A. Carbon pricing in New Zealand: implications for public health. N Z Med J. 2009;122(1290):105-15.
115. Dwivedi SL, Lammerts van Bueren ET, Ceccarelli S, Grando S, Upadhyaya HD, Ortiz R. Diversifying Food Systems in the Pursuit of Sustainable Food Production and Healthy Diets. Trends in Plant Science. 2017;22(10):842-56.
116. Egger G. Dousing our inflammatory environment(s): Is personal carbon trading an option for reducing obesity - And climate change? Obesity reviews : an official journal of the International Association for the Study of Obesity. 2008;9:456-63.

117. Elmqvist T, Setälä H, Handel SN, van der Ploeg S, Aronson J, Blignaut JN, et al. Benefits of restoring ecosystem services in urban areas. Current Opinion in Environmental Sustainability. 2015;14:101-8.
118. Williams S, Nitschke M, Tucker G, Bi P. Extreme Heat Arrangements in South Australia: an assessment of trigger temperatures. Health promotion journal of Australia : official journal of Australian Association of Health Promotion Professionals. 2011;22 Spec No:S21-7.
119. Fisk WJ. Review of some effects of climate change on indoor environmental quality and health and associated no-regrets mitigation measures. Building and Environment. 2015;86:70-80.
120. Gulyas BZ, Edmondson JL. Increasing City Resilience through Urban Agriculture: Challenges and Solutions in the Global North. Sustainability. 2021;13(3):1465.
121. Halsnæs K, Some S, Pathak M. Beyond synergies: understanding SDG trade-offs, equity and implementation challenges of sectoral climate change mitigation options. Sustainability Science. 2024;19(1):35-49.
122. Holmner Å, Joacim R, Nawi N, and Nilsson M. Climate change and eHealth: a promising strategy for health sector mitigation and adaptation. Global Health Action. 2012;5(1):18428.
123. Hu Y, Cheng J, Liu S, Tan J, Yan C, Yu G, et al. Evaluation of climate change adaptation measures for childhood asthma: A systematic review of epidemiological evidence. Science of The Total Environment. 2022;839:156291.
124. Issac S, Shultz JM, Espinel Z, Alvarez G, Shapiro LT. In the Cone of Concern: Preparing Stroke Survivors for Extreme Hurricanes. Am J Phys Med Rehabil. 2023.
125. Jay O, Capon A, Berry P, Broderick C, de Dear R, Havenith G, et al. Reducing the health effects of hot weather and heat extremes: from personal cooling strategies to green cities. The Lancet. 2021;398(10301):709-24.
126. Jennings N, Fecht D, De Matteis S. Mapping the co-benefits of climate change action to issues of public concern in the UK: a narrative review. The Lancet Planetary Health. 2020;4(9):e424-e33.
127. Jones SJ. If electric cars are the answer, what was the question? British Medical Bulletin. 2019;129(1):13-23.
128. Kardan M, Akter T, Iqbal M, Tcymbal A, Messing S, Gelius P, et al. Cycling in older adults: a scoping review. Frontiers in Sports and Active Living. 2023;Volume 5 - 2023.
129. Kim Y, Oka K, Kawazu EC, Ng CFS, Seposo X, Ueda K, et al. Enhancing health resilience in Japan in a changing climate. The Lancet Regional Health – Western Pacific. 2023;40.
130. Kime S, Jacome V, Pellow D, Deshmukh R. Evaluating equity and justice in low-carbon energy transitions. Environmental Research Letters. 2023;18(12):123003.
131. Lake IR, Hooper L, Abdelhamid A, Bentham G, Boxall ABA, Draper A, et al. Climate Change and Food Security: Health Impacts in Developed Countries. Environmental Health Perspectives. 2012;120(11):1520-6.
132. Lake IR, Barker GC. Climate Change, Foodborne Pathogens and Illness in Higher-Income Countries. Current Environmental Health Reports. 2018;5(1):187-96.

133. Leder K, Sinclair MI, McNeil JJ. Water and the environment: a natural resource or a limited luxury? Med J Aust. 2002;177(11-12):609-13.
134. Lenzer B, Rupprecht M, Hoffmann C, Hoffmann P, Liebers U. Health effects of heating, ventilation and air conditioning on hospital patients: a scoping review. BMC Public Health. 2020;20(1):1287.
135. Linares C, Díaz J, Negev M, Martínez GS, Debono R, Paz S. Impacts of climate change on the public health of the Mediterranean Basin population - Current situation, projections, preparedness and adaptation. Environmental Research. 2020;182:109107.
136. Lowe M. Obesity and climate change mitigation in Australia: overview and analysis of policies with co - benefits. Australian and New Zealand Journal of Public Health. 2014;38(1):19-24.
137. Lowe D, Ebi KL, Forsberg B. Heatwave Early Warning Systems and Adaptation Advice to Reduce Human Health Consequences of Heatwaves. International Journal of Environmental Research and Public Health. 2011;8(12):4623-48.
138. Luyten A, Winkler MS, Ammann P, Dietler D. Health impact studies of climate change adaptation and mitigation measures – A scoping review. The Journal of Climate Change and Health. 2023;9:100186.
139. Magkos F, Tetens I, Bügel SG, Felby C, Schacht SR, Hill JO, et al. A Perspective on the Transition to Plant-Based Diets: a Diet Change May Attenuate Climate Change, but Can It Also Attenuate Obesity and Chronic Disease Risk? Advances in Nutrition. 2020;11(1):1-9.
140. Mailloux NA, Henegan CP, Lsoto D, Patterson KP, West PC, Foley JA, et al. Climate Solutions Double as Health Interventions. International Journal of Environmental Research and Public Health. 2021;18(24):13339.
141. Markkanen S, Anger-Kraavi A. Social impacts of climate change mitigation policies and their implications for inequality. Climate Policy. 2019;19(7):827-44.
142. McMurtry RY, Krogh CM. Diagnostic criteria for adverse health effects in the environs of wind turbines. JRSM Open. 2014;5(10):2054270414554048.
143. Milner J, Davies M, Wilkinson P. Urban energy, carbon management (low carbon cities) and co-benefits for human health. Current Opinion in Environmental Sustainability. 2012;4(4):398-404.
144. Nieuwenhuijsen MJ, Khreis H. Car free cities: Pathway to healthy urban living. Environment International. 2016;94:251-62.
145. Nori-Sarma A, Wellenius GA. Human Health and Well-being in a Warming World. Milbank Q. 2023;101(S1):99-118.
146. O'Neill MS, Carter R, Kish JK, Gronlund CJ, White-Newsome JL, Manarolla X, et al. Preventing heat-related morbidity and mortality: new approaches in a changing climate. Maturitas. 2009;64(2):98-103.
147. Oliveira JA, Doll CN, Siri J, Dreyfus M, Farzaneh H, Capon A. Urban governance and the systems approaches to health-environment co-benefits in cities. Cad Saude Publica. 2015;31 Suppl 1:25-38.
148. Perera F, Ashrafi A, Kinney P, Mills D. Towards a fuller assessment of benefits to children's health of reducing air pollution and mitigating climate change due to fossil fuel combustion. Environmental Research. 2019;172:55-72.

149. Picetti R, Juel R, Milner J, Bonell A, Karakas F, Dangour AD, et al. Effects on child and adolescent health of climate change mitigation policies: A systematic review of modelling studies. Environmental Research. 2023;238:117102.
150. Maria Rodgers ORMRKMPZ. Plants of place: justice through (re)planting Aotearoa New Zealand’s urban natural heritage. Architecture_MPS. 2023;25(1).
151. Quam VGM, Rocklöv J, Quam MBM, Lucas RAI. Assessing Greenhouse Gas Emissions and Health Co-Benefits: A Structured Review of Lifestyle-Related Climate Change Mitigation Strategies. Int J Environ Res Public Health. 2017;14(5).
152. Sharifi A, Pathak M, Joshi C, He B-J. A systematic review of the health co-benefits of urban climate change adaptation. Sustainable Cities and Society. 2021;74:103190.
153. Sharma A, Shiwang J, Lee A, Peng W. Equity implications of electric vehicles: A systematic review on the spatial distribution of emissions, air pollution and health impacts. Environmental Research Letters. 2023;18(5):053001.
154. Shaw C, Hales S, Howden-Chapman P, Edwards R. Health co-benefits of climate change mitigation policies in the transport sector. Nature Climate Change. 2014;4(6):427-33.
155. Shonkoff SB, Morello-Frosch R, Pastor M, Sadd J. The climate gap: environmental health and equity implications of climate change and mitigation policies in California—a review of the literature. Climatic Change. 2011;109(1):485-503.
156. Sin DD, Doiron D, Agusti A, Anzueto A, Barnes PJ, Celli BR, et al. Air pollution and COPD: GOLD 2023 committee report. European Respiratory Journal. 2023;61(5):2202469.
157. Smith KR, Frumkin H, Balakrishnan K, Butler CD, Chafe ZA, Fairlie I, et al. Energy and human health. Annu Rev Public Health. 2013;34:159-88.
158. Wang L, Zhong B, Vardoulakis S, Zhang F, Pilot E, Li Y, et al. Air Quality Strategies on Public Health and Health Equity in Europe-A Systematic Review. Int J Environ Res Public Health. 2016;13(12).
